# Integrative cross‐tissue and spatially resolved single‐cell profiling uncovers tumour‐educated inflammatory remodelling of tissue‐resident macrophage ecosystem with immunotherapeutic prognostic significance in pan‐cancer

**DOI:** 10.1002/ctm2.70608

**Published:** 2026-02-08

**Authors:** Weikai Wang, Zibin Chen, Yuhan Huang, Zhihao Hu, Peixin Zhu, Zhuoli Huang, Jingzhi Lv, Ziru Liao, Yuhui Zheng, Chen Wei, Baibing Guan, Yin Zeng, Xinyue Zhu, Yafei Yang, Guibo Li, Xin Jin, Xi Chen, Xiao Yang, Zikun Ma, Jianhua Yin

**Affiliations:** ^1^ College of Life Sciences University of Chinese Academy of Sciences Beijing China; ^2^ State Key Laboratory of Genome and Multi‐omics Technologies BGI Research Shenzhen China; ^3^ Department of Urology; Sun Yat‐sen University Cancer Center; State Key Laboratory of Oncology in South China Collaborative Innovation Center for Cancer Medicine Guangzhou China; ^4^ BGI College & Henan Institute of Medical and Pharmaceutical Sciences Zhengzhou University Zhengzhou China; ^5^ BGI Research Chongqing China; ^6^ School of Biology and Biological Engineering South China University of Technology Guangzhou China; ^7^ Zhangzhou Municipal Hospital Affiliated to Fujian Medical University Zhangzhou China; ^8^ Department of Ultrasound, Peking Union Medical College Hospital Chinese Academy of Medical Sciences and Peking Union Medical College Beijing China; ^9^ Shenzhen Proof‐of‐Concept Center of Digital Cytopathology BGI Research Shenzhen China; ^10^ Shanxi Medical University ‐ BGI Collaborative Center for Future Medicine Shanxi Medical University Taiyuan China

**Keywords:** machine learning, pan‐cancer, single‐cell RNA sequencing, spatial transcriptome sequencing, tissue‐resident macrophages, tumour microenvironment

## Abstract

**Background:**

Tissue‐resident macrophages (TRMs) exhibit dual roles in tumor progression, yet their functional reprogramming within the tumor microenvironment (TME) remains a critical unresolved question.

**Methods:**

We integrated single‐cell and spatial transcriptomics from a pan‐cancer atlas of 1.39 million cells across five malignancies with 2,318 bulk RNA‐seq samples to investigate macrophage states. A TRM inflammatory remodeling signature (TIR‐Sig) was developed for clinical biomarker validation.

**Results:**

We identified a conserved inflammatory TRM subtype (iTRM) characterized by CXCL8/IL1B/IL6 co‐expression that correlates with poor clinical outcomes. Crucially, both TRMs and monocyte‐derived tumor‐associated macrophages (Mono‐TAMs) underwent convergent differentiation into functionally similar inflammatory phenotypes, establishing iTRM as a universal tumor‐educated state. Further integration analysis revealed an iTRM‐enriched TME subtype which featured coordinated infiltration of neutrophils and cancer‐associated fibroblasts (CAFs), forming a ‘cold tumor’ ecosystem associated with immune checkpoint blockade (ICB) resistance and poor prognosis. The derived TRM inflammatory remodeling signature (TIR‐Sig) demonstrated dual clinical utility: it predicted patient survival (HR = 19.86, *p* < .001) and stratified ICB responders (AUC = .706).

**Conclusion:**

This study establishes phenotypic links between tissue‐resident and recruited macrophages through inflammatory reprogramming within TME, provides a unifying framework for pan‐cancer macrophage plasticity in TME, delivers a clinically actionable biomarker suite (TIR‐Sig), and provides potential therapeutic targets for TME remodeling.

**Key points:**

- Cross‐tissue single‐cell atlas of tissue‐resident macrophages (TRMs).
- Identification of conserved inflammatory TRM phenotype (iTRM) in pan‐cancer.
- Dynamic convergence of TRM and monocyte‐derived macrophage lineages.
- TRM inflammatory remodelling signature (TIR‐Sig) with clinical potential.

## Graphical abstract

Pan‐cancer analysis reveals an inflammatory phenotype remodeling of tissue‐resident macrophages (iTRM) in the tumor microenvironment, which is associated with immunosuppression. Tissue‐resident macrophages and monocyte‐derived macrophages exhibit convergent differentiation toward a similar inflammatory phenotype. iTRM‐enriched ecosystems are associated with poorer prognosis and increased resistance to immune checkpoint therapy, prompting the development of the TIR‐Sig biomarker for both prognostic assessment and treatment prediction.

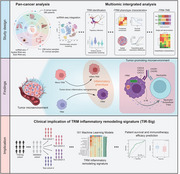

## 1 INTRODUCTION

The tumour microenvironment (TME) has increasingly been recognised as a complex ecosystem comprising diverse cell types and intricate interactions.^1^ The dynamic interplay between tumour cells, stromal cells and immune cells profoundly influences tumour development, progression, prognosis and therapeutic responses.^2^, ^3^, ^4^ For instance, immune checkpoint inhibitors (ICIs) demonstrated efficacy by reinvigorating anti‐tumour immunity, but their success is often limited by the immunosuppressive nature of the TME.^5^, ^6^ Within TME, tumour‐associated macrophages (TAMs) play a crucial, yet complex role.^7^, ^8^ TAMs are derived from peripheral monocytes or tissue‐resident macrophages (TRMs).^9^ Traditionally, research has focused on monocyte‐derived TAM (Mono‐TAMs), which are often associated with immunosuppressive or pro‐tumourigenic phenotypes that accelerate disease progression.^10^, ^11^, ^12^, ^13^ However, accumulating evidence emphasises the critical role of TRM‐derived TAM (TRM‐TAMs). This is reflected in divergent clinical outcomes: Phase II trials targeting monocyte recruitment pathways, including the anti‐CCL2 antibody carlumab in prostate cancer^14^ and the CSF1R inhibitor PLX3397 in glioblastoma (GBM),^15^ have failed, whereas therapies broadly modulating TAM populations such as CD40 agonists or Toll‐like receptor (TLR) 8 activators demonstrate efficacy in pancreatic and squamous cell carcinoma of the head and neck.^16^ Consequently, targeting TRM‐TAMs is necessary.

TRMs are self‐renewing cells originating from yolk‐sac progenitors that colonise tissues during organogenesis.^17^ Similar to peripheral macrophages, they exhibit phagocytic capacity and immunoregulatory functions, typically maintaining a homeostatic immunosuppressive phenotype.^18^, ^19^ Recent studies demonstrate that tumour cells hijack TRM within TME to drive immunosuppression and promote disease progression. In non‐small cell lung cancer (NSCLC), TRM promotes epithelial–mesenchymal transition (EMT) and invasiveness in tumour cells, while simultaneously inducing a potent regulatory T cell (Treg) response that enables tumour cells to evade adaptive immune destruction.^20^ Parallelly, in pancreatic ductal adenocarcinoma, proliferating TRM drives fibrosis and immunosuppression while reducing gemcitabine uptake through elevated deoxycytidine (dC) production and suppressed dC kinase expression, thereby compromising chemotherapeutic efficacy.^21^ Furthermore, VSIG4+ TRM impairs CD8+ T cell immune function through VSIG4‐mediated receptor engagement and IL‐11 paracrine signalling in multiple solid tumours.^22^ These findings indicate that tumours could remodel TRM from tissue homeostasis guardians into immunosuppressive collaborators. Nevertheless, tumour‐specific patterns of TRM phenotypic remodelling and the functional relationship between TRM‐TAM and Mono‐TAM remain unclear, particularly whether these populations evolve independently or undergo phenotypic convergence. Resolving these questions is essential for developing effective macrophage‐targeted therapies.

To address these gaps, we performed an integrative cross‐tissue single‐cell transcriptomic analysis of five solid tumours (lung adenocarcinoma [LUAD], hepatocellular carcinoma [HCC], colorectal cancer [CRC], GBM, skin cutaneous melanoma [SKCM]), encompassing over 1.39 million cells, to systematically dissect TRM heterogeneity and plasticity. Our analysis revealed a conserved inflammatory phenotype remodelling of TRMs across cancers. We identified a pro‐inflammatory TRM subset (iTRM) expressing high levels of CXCL8, IL‐1B and IL‐6, exhibiting potent neutrophil recruitment capacity and significantly correlating with poor prognosis in multiple cancers. Trajectory analysis revealed that iTRM and Mono‐TAM, despite their distinct developmental origins, converge towards an inflammatory state in the TME, with accelerated TRM inflammatory phenotype conversion and dynamic reprogramming of extracellular matrix (ECM) remodelling pathways in tumour tissues. Furthermore, we identified TME subtypes highly associated with iTRM phenotypes and functions, exhibiting unique associations with immune checkpoint inhibition (ICI) non‐response. Based on the iTRM and TME characteristics, we constructed a TRM inflammatory remodelling signature (TIR‐Sig), demonstrating precise predictive power for patient survival and immune therapy response in independent cohorts, providing new strategies and tools for targeted therapy. This study indicates  iTRM as a conserved ‘tumour‐educated’ macrophage state across cancer types, bridging the pro‐tumourigenic functions of tissue‐resident and monocyte‐derived macrophages (MDMs) through phenotypic remodelling, providing insights into macrophage function and phenotypic changes in the TME, suggesting that targeting TRM phenotypic transformation may be a novel therapeutic strategy across cancers.

## 2 RESULT

### 2.1 Large‐scale profiling of TRMs in pan‐cancers

**FIGURE 1:**
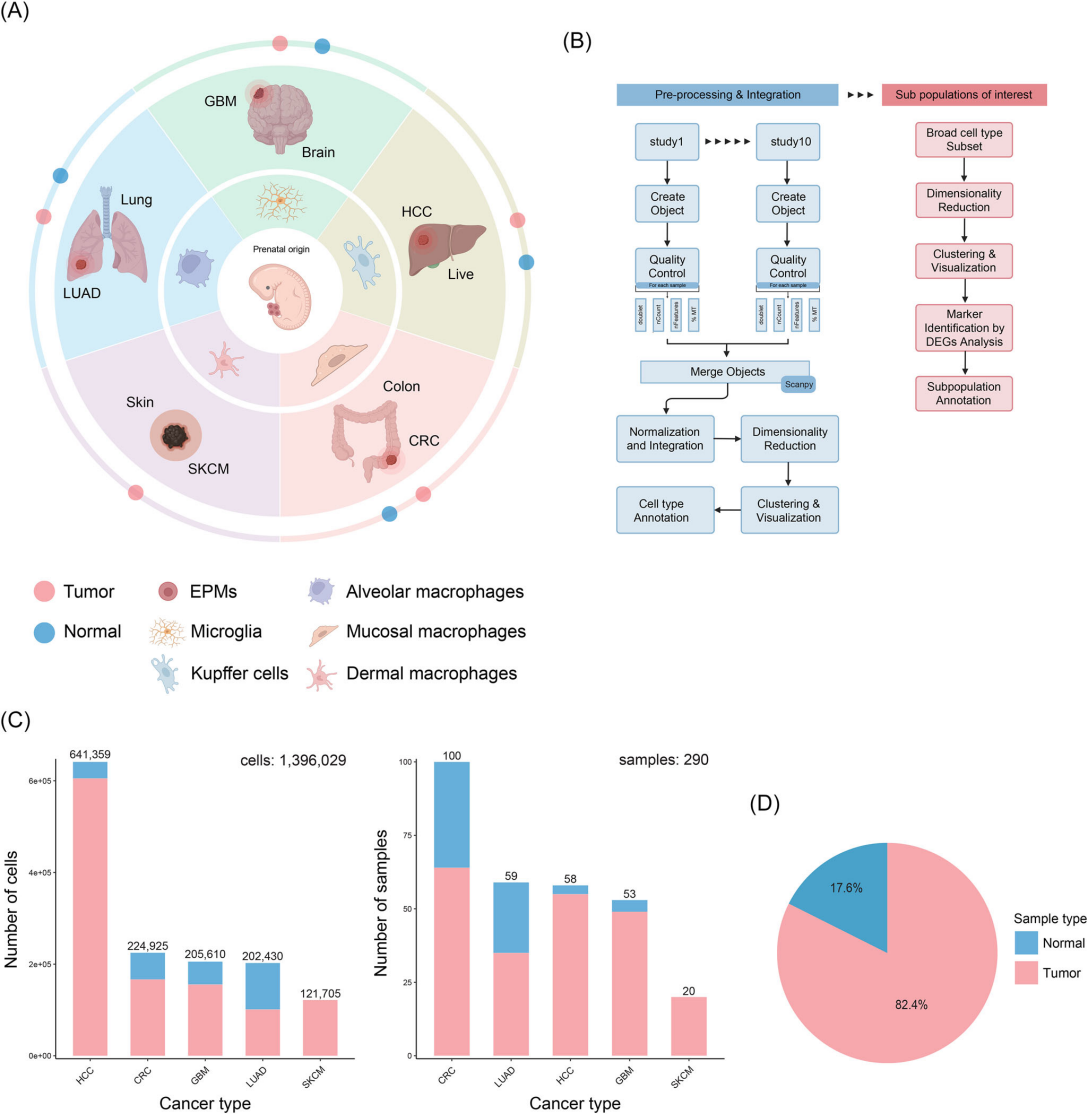
Single‐cell atlas landscape of tissue‐resident macrophages (TRMs) across tumour and adjacent normal tissues. (A) Schematic representation of the study design. We constructed a pan‐cancer TRM atlas by integrating 10 published single‐cell datasets from five tissues harbouring yolk sac‐derived TRMs. (B) Analytical workflow encompassing data curation, batch effect correction, unsupervised clustering and functional annotation. (C) Bar plots quantifying cell counts (left) and sample sizes (right) per cancer type, colour‐coded by tissue type (tumour vs. adjacent normal). (D) Pie chart showing proportional distribution of sample origins (tumour vs. adjacent normal).

To elucidate the characteristics of TAM subpopulations, especially TRM‐TAM, across diverse tissue types, we conducted an integrated cross‐tissue analysis of single‐cell datasets from 10 public studies encompassing five TRM‐enriched malignancies^23^, ^24^, ^25^, ^26^, ^27^, ^28^, ^29^, ^30^, ^31^, ^32^(Figure 1A). This cohort included CRC, HCC, LUAD, GBM and SKCM. The non‐pathological target organs of these malignancies contain definitive yolk sac‐derived TRM populations.^33^ This enabled comparative analysis of developmentally distinct TAM subsets, providing a comprehensive view of macrophage behaviour across divergent TMEs. Following stringent quality control measures and batch effect correction (Figures S1C,E and S2A,C), we acquired a total of 1 396 029 single‐cell transcriptomes from 290 samples (CRC: *n* = 76, HCC: *n* = 55, LUAD: *n* = 52, GBM: *n* = 43, SKCM: *n* = 17). The majority of these cells (82.4%) originated from tumour tissues, while the remaining 17.6% were derived from cancer‐adjacent normal tissues (Figure 1C). This distribution allowed for a comparative analysis of macrophage phenotypes in malignant versus non‐malignant states.

We performed initial analysis using dimensionality reduction and unsupervised clustering, classifying cells into seven major types based on canonical markers and transcriptomic signatures^34^, ^35^ (Figure S1A,B,D). We then focused on the macrophage compartment, specifically tumour‐associated TRM (TRM‐TAM). We identified TRM‐TAM subpopulations distinct from Mono‐TAM in each tissue, based on the expression of tissue‐specific TRM markers and unsupervised trajectory analysis (Figure S3B). Tissue‐specific TRM subpopulations exhibited high expression of corresponding tissue‐resident marker genes^33^, ^36^ (e.g., TMEM119 in microglia, FABP1 in alveolar TRMs, CD5L in Kupffer cells, LYVE1 in intestinal TRMs, FOLR2 in dermal TRMs) (Figure S3A). This confirmed the presence of both TRM‐TAMs and Mono‐TAMs within our dataset, allowing us to study their unique characteristics and potential interactions.

To further validate the origin reliability of the identified TRM‐TAM and Mono‐TAM subpopulations, we collected embryonic‐origin signatures and monocyte‐derived signatures of macrophages from published studies (Table S1) and characterised them across TAM subpopulations. We observed significant negative correlations between embryonic‐origin and monocyte‐derived signatures in all TAM subpopulations across tissues (Figure S4A), reflecting the mutually exclusive nature of these signatures in macrophage physiological functions. Moreover, TRM‐TAM subpopulations consistently exhibited significantly higher embryonic‐origin signatures than Mono‐TAM subpopulations in all tissues (Figure S4B), while Mono‐TAM subpopulations showed conversely higher monocyte‐derived signatures than TRM‐TAMs. These results support the strong association of our identified TRM‐TAM subpopulations with bona fide embryonically derived TRMs.

### 2.2 Single‐cell transcriptome characterisation of TRM‐TAM

**FIGURE 2:**
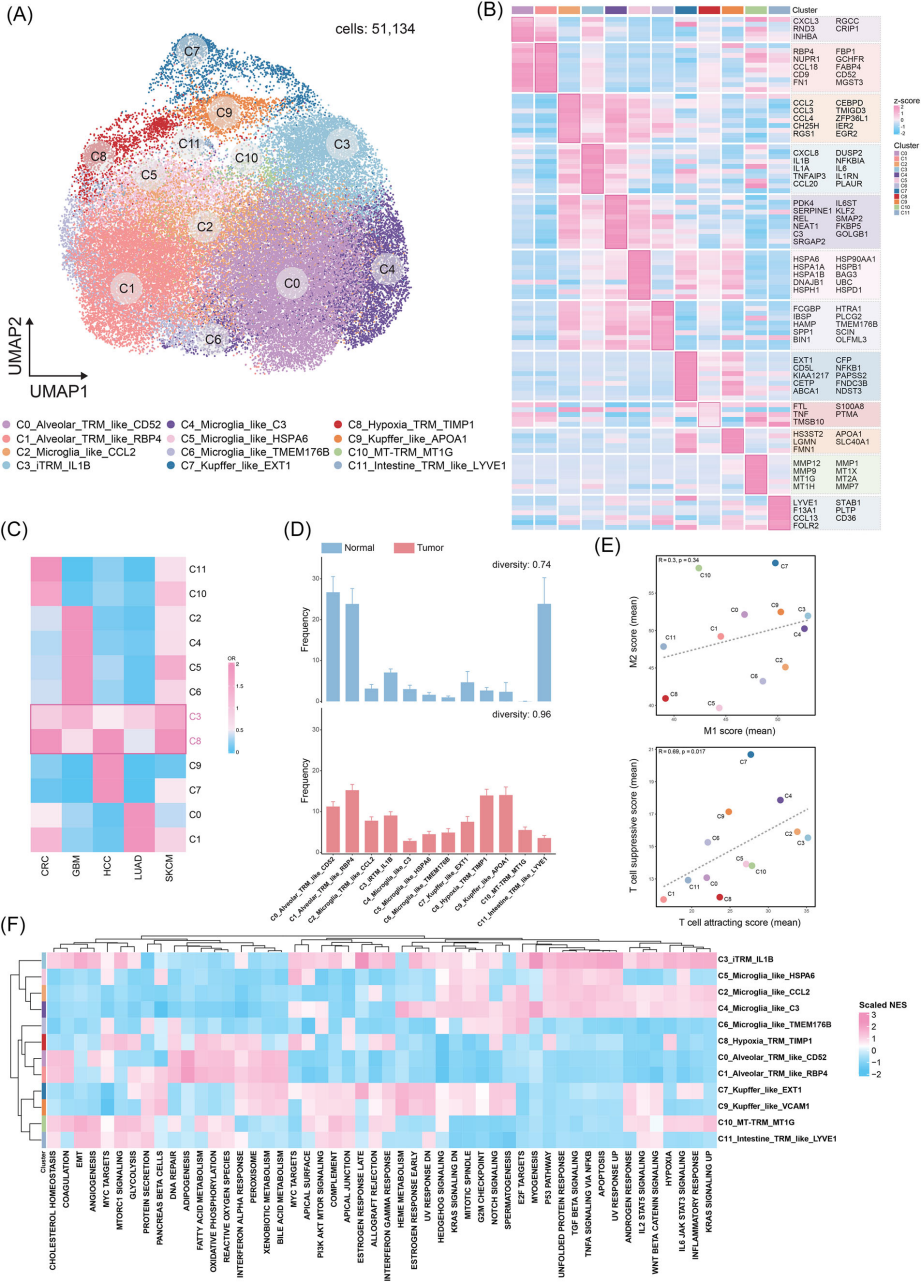
Characterisation of tissue‐resident macrophages (TRM) in the tumour microenvironment. (A) UMAP visualisation of TRM‐TAM subpopulations. (B) Heatmap of TRM‐TAM subpopulation‐specific differentially expressed genes (DEGs). (C) Odds ratio (OR) heatmap for tissue distribution bias of TRM subpopulations (OR < .5 indicates significant depletion in the corresponding tissue; rows and columns hierarchically clustered by cosine distance). (D) Bar plot comparing TRM subpopulation composition between normal and tumour tissues (mean ± SD). Tissue‐specific diversity is quantified by the Shannon equitability index (higher values indicate more balanced frequencies across subpopulations). (E) Functional polarisation analysis of TRM subpopulations: Scatter plots showing (left) average M1‐polarised (pro‐inflammatory) versus M2‐polarised (anti‐inflammatory) scores and (right) average T cell‐attracting versus T cell‐suppressing scores. (F) Heatmap of Hallmark pathway gene set enrichment analysis (GSEA) for 12 TRM‐TAM subpopulations.

To further characterise these TRM‐TAM subpopulations, we performed subclustering and identified distinct functional states based on their unique gene expression profiles. To this end, after correcting for batch effects (Figure S2B,D–F), we re‐clustered the TRM‐TAM compartment, identifying 12 distinct cross‐tissue TRM‐TAM subpopulations (51 134 cells) (Figure 2A). Classical pan‐macrophage markers such as CD68, CD163 and C1QA were highly expressed in all TRM‐TAM subpopulations^37^, ^38^ (Figure S5A). In contrast, the expression levels of monocyte or dendritic cell markers such as VCAN, S100A8, FCN1, CD1C, CLEC9A and CCR7 was low^39^, ^40^ (Figure S5B). To further characterise each TRM‐TAM subpopulation, we examined the expression of their top marker genes (Figure 2B).

Specifically, C0_Alveolar_TRM_like_CD52 and C1_Alveolar_TRM_like_RBP4 subsets are the two most abundant TRM‐TAM subpopulations, comprising 13 390 and 8128 cells, respectively. These clusters are predominantly derived from lung tissue and likely represent alveolar macrophages^41^ (AMs). C0_Alveolar_TRM_like_CD52 and C1_Alveolar_TRM_like_RBP4 both exhibit up‐regulated expression of FABP4 and RBP4, indicating active fatty acid uptake and retinol transport activities, consistent with the lipid metabolic properties of AMs.^42^, ^43^, ^44^ Notably, the C0_Alveolar_TRM_like_CD52 subpopulation shows elevated expression of INHBA, a driver of activin A‐mediated fibrotic remodelling, suggesting its potential role in balancing alveolar repair and fibrosis,^45^, ^46^ while the C1_Alveolar_TRM_like_RBP4 subpopulation may establish an immunosuppressive TME through CCL18‐dependent recruitment of Tregs.^47^

C2_Microglia_like_CCL2, C4_Microglia_like_C3, C5_Microglia_like_HSPA6 and C6_Microglia_like_TMEM176B subpopulations specifically express microglial markers (TMEM119, TREM2, P2RY12).^48^, ^49^ C2_Microglia_like_CCL2 demonstrates up‐regulated chemokines CCL2/CCL3/CCL4, reflecting enhanced monocyte recruitment capacity, aligning with previously reported microglial functional adaptations during neuroinflammation.^50^ C4_Microglia_like_C3 elevates KLF2 and complement C3 expression, indicative of active endothelial crosstalk^51^, ^52^ and complement activation,^53^ potentially implicating its involvement in blood–brain barrier (BBB) regulation.^54^ C5_Microglia_like_HSPA6 exhibits broad up‐regulation of heat shock protein genes (HSPA6/HSPA1A/HSP90AA1), indicative of a proteostasis‐adapted state that mitigates proteotoxic stress within the TME.^55^, ^56^, ^57^ This molecular signature aligns with microglia‐like cells’ capacity to survive under TME‐specific pressures, analogous to their role in countering protein misfolding stress driven by tumour‐derived factors such as lactate overload and hypoxia‐induced proteome instability.^58^, ^59^ The C6_Microglia_like_TMEM176B exhibits significant overexpression of the lysosomal acidification regulator TMEM176B and osteopontin (SPP1), indicative of enhanced phagolysosomal activation^60^ and potentiated ECM degradation.^61^ This molecular signature parallels the immunosuppressive properties of SPP1+ TAM.^57^

C3_iTRM_IL1B exhibits a pro‐inflammatory phenotype characterised by significant up‐regulation of IL‐1B, IL‐6, CXCL8, PLAUR and NF‐κB pathway regulators (TNFAIP3, NFKBIA).^62^ The overexpression of CXCL8, a key neutrophil chemoattractant, further suggests its potential to recruit neutrophils and collaboratively shape the tumour immune microenvironment under inflammatory conditions.^63^ C8_Hypoxia_TRM_TIMP1 co‐expresses TIMP1 and FTL, a signature commonly observed in hypoxia‐adapted TAMs, likely reflecting adaptation to oxygen‐deprived tumour core regions.^64^, ^65^ However, this subset displays a naïve‐like transcriptional profile with limited functional specificity.

Both C7_Kupffer_like_EXT1 and C9_Kupffer_like_APOA1 subsets highly express Kupffer cell signature genes (VSIG4, CD5L, MARCO).^66^ C7_Kupffer_like_EXT1 up‐regulates liver sinusoid‐associated genes EXT1 (heparan sulphate synthesis), CFP (complement regulation) and ABCA1 (cholesterol efflux), implicating its roles in liver‐specific immune modulation, sinusoidal endothelial adhesion and lipid metabolism.^67^, ^68^, ^69^ In contrast, C9_Kupffer_like_APOA1 specifically overexpresses APOA1 and LGMN, implicating its functional involvement in high‐density lipoprotein assembly^70^ and lysosomal antigen processing.^71^ Furthermore, previous studies indicate that elevated APOA1 expression drives M2‐polarisation^72^ while orchestrating lipid metabolic reprogramming,^73^ thereby facilitating the establishment of immunosuppressive homeostasis.

C10_MT‐TRM_MT1G shows marked enrichment of metallothionein genes (MT1G/MT1X/MT2A) coupled with elevated matrix metalloproteinases (MMP1/MMP9), potentially mediating intestinal barrier defense^74^ and stromal remodelling.^75^ C11_Intestine_TRM_like_LYVE1 closely resembles the previously reported LYVE1^+^FOLR2^+^ intestine macrophage subset,^76^ overexpressing LYVE1 (lymphatic endothelial interaction), FOLR2 (folate uptake and tissue homeostasis), STAB1 (apoptotic cell clearance and ECM remodelling), F13A1 (fibrin stabilisation) and PLTP/CD36 (lipid metabolism regulation). These features collectively highlight its multifaceted roles in tissue repair, lipid metabolism and immunomodulation.

We further characterised the function of these 12 TRM‐TAMs (Figure 2F). C0_Alveolar_TRM_like_CD52 and C1_Alveolar_TRM_like_RBP4 exemplify metabolic specialisation within the pulmonary niche. While both clusters engage lipid metabolism, C0 demonstrates reliance on oxidative phosphorylation (NES: 1.34) that supports sustained energy production essential for AM longevity.^77^ In contrast, C1 exhibits fatty acid metabolism (NES: 1.57) coupled with RBP4‐driven retinol transport. This functional involvement in dynamic surfactant composition regulation represents a critical mechanism for maintaining lung compliance.^78^ This metabolic bifurcation may reflect compartmentalised functions: C0 stabilising lipid reservoirs and C1 facilitating surfactant recycling through retinoid‐mediated transcriptional regulation. C2_Microglia_TRM_like_CCL2 exhibited moderate enrichment in inflammatory signalling pathways (TNFA/NFKB NES: 1.32; INFLAMMATORY RESPONSE NES: 1.06), which may amplify inflammatory states in the TME through CCL2‐mediated monocyte recruitment and NF‐κB‐driven release of pro‐inflammatory factors (e.g., TNF‐α). Conversely, C5_Microglia_like_HSPA6 and C6_Microglia_like_TMEM176B counterbalanced inflammation through distinct mechanisms: HSPA6 resolved proteotoxic stress via the unfolded protein response (NES: 1.25), while TMEM176B suppressed NF‐κB signalling (NES: −.99), collectively representing counteractive regulatory interactions among TRM subsets in the TME. Notably, C4_Microglia_like_C3 exhibits elevated C3 expression alongside paradoxical complement pathway suppression (NES: −.93). This apparent decoupling may co‐occur with EMT (NES: .66), potentially representing a coordinated strategy to limit synaptic loss during development while facilitating tissue repair.^79^, ^80^ Such a physiological program could be co‐opted to support microenvironmental remodelling in gliomas.^81^

C3_iTRM_IL1B may represent a key pro‐iTRM‐TAM population characterised by coordinated activation of three core pathways: interferon‐gamma (IFN‐γ) response (NES: 2.39), TNF‐α/NF‐κB signalling (NES: 1.75) and IL‐6/JAK–STAT3 (NES: 1.48). These pathways collectively appear to contribute to enhanced IL‐1β production,^82^ potentially driving angiogenesis,^83^ neutrophil extracellular trap (NET) formation^84^ and Th17 polarisation.^85^ This inflammatory activation could promote tumour progression via multiple mechanisms. Sustained inflammation mediated by chronic IL‐1β secretion may recruit immunosuppressive myeloid cells and remodel vascular niches.^86^ Concurrently, STAT3 activation might suppress cytotoxic T‐cell function while expanding Tregs, reinforcing an immunosuppressive TME.^87^ Additionally, IL‐1β‐induced NET formation could facilitate metastasis by trapping circulating tumour cells (CTCs) and reducing immune surveillance.^88^ C8_Hypoxia_TRM_TIMP1 exhibits coordinated up‐regulation of glycolysis (NES: 1.04) and hypoxia signalling (NES: 1.03), indicating its potential enrichment and adaptation within ischemic tumour cores.^89^, ^90^

C7_Kupffer_like_EXT1 may promote fibrin‐rich microthrombi formation, potentially through coordinated activation of coagulation pathways (NES: 1.25) and complement signalling (NES: .96).^91^, ^92^ This process could enhance CTC adhesion to vascular endothelia,^93^ while possibly contributing to immune evasion in metastatic niches by reducing NK cell‐mediated surveillance.^94^ In parallel, C9_Kupffer_like_APOA1 may exploit APOA1‐mediated cholesterol efflux (cholesterol homeostasis NES: .96), potentially depleting membrane lipid rafts in T cells, which could contribute to impaired anti‐tumour TCR signalling.^95^, ^96^ This metabolic adaptation appears to be leveraged by CRC liver metastases as a potential mechanism for adaptive immune evasion.^82^

C10_MT‐TRM_MT1G and C11_Intestine_TRM_like_LYVE1 exhibit activation of mTORC1 signalling (NES: 1.70 and 1.39, respectively). mTORC1 is a master regulator of cellular metabolism, growth and protein synthesis. In macrophages, mTORC1 signalling has been implicated in promoting pro‐reparative functions, which could potentially support processes like epithelial regeneration and angiogenesis within the TME.^97^, ^98^ The high expression of MT1G in C10_MT‐TRM_MT1G suggests a role in metal ion homeostasis and protection against oxidative stress. This function might contribute to maintaining a favourable niche for intestinal stem cells by scavenging toxic metals or reactive oxygen species in their vicinity.^99^ The expression of LYVE1 on C11_Intestine_TRM_like_LYVE1 macrophages implies a potential spatial association with lymphatic vessels and/or a capacity for hyaluronan uptake. Such positioning or interaction might facilitate the surveillance of tissue antigens or the recruitment/guidance of immune cells (e.g., dendritic cells or T cells) towards lymphatic vessels during inflammatory responses, thereby contributing to immune cell trafficking between the tissue and draining lymph nodes.^100^, ^101^, ^102^

Furthermore, considering the signature markers of M1/M2 macrophages and their potential effects on T cells, we observed that most TRM‐TAMs subpopulations exhibited co‐enrichment of both M1 and M2 features, particularly C3_iTRM_IL1B, C4_Microglia_like_C3 and C9_Kupffer_like_APOA1. In contrast, C10_MT‐TRM_MT1G and C7_Kupffer_like_EXT1 displayed a stronger bias towards an M2‐like phenotype, whereas C2_Microglia_like_CCL2 and C6_Microglia_like_TMEM176B skewed towards an M1‐like polarisation. Notably, C8_Hypoxia_TRM_TIMP1 showed minimal activation of either M1 or M2 signatures. Functionally, C7_Kupffer_like_EXT1 demonstrated enhanced T cell‐suppressive capacity, while C2_Microglia_like_CCL2 and C3_iTRM_IL1B were associated with robust T cell recruitment (Figure 2E).

Owing to the inherent tissue tropism of TRM, we next investigated the cross‐tissue distribution of each TRM subpopulation. We found that most TRM‐TAMs subpopulations retained strong tissue specificity, remaining predominantly localised to their primary tissues even after integrative re‐clustering analysis. Notably, C3_iTRM_IL1B and C8_Hypoxia_TRM_TIMP1 exhibited a ubiquitous presence across all tissues, suggesting their potential utility for identifying shared functional or molecular features of TRM‐TAMs in pan‐tissue contexts (Figure 2C). Furthermore, comparative analysis revealed significant shifts in the proportional abundances of TRM‐TAMs subpopulations in tumour tissues relative to normal counterparts (Figure 2D), implying substantial remodelling of TRM‐TAMs under tumour microenvironmental pressures.

### 2.3 Identification of iTRMs

**FIGURE 3:**
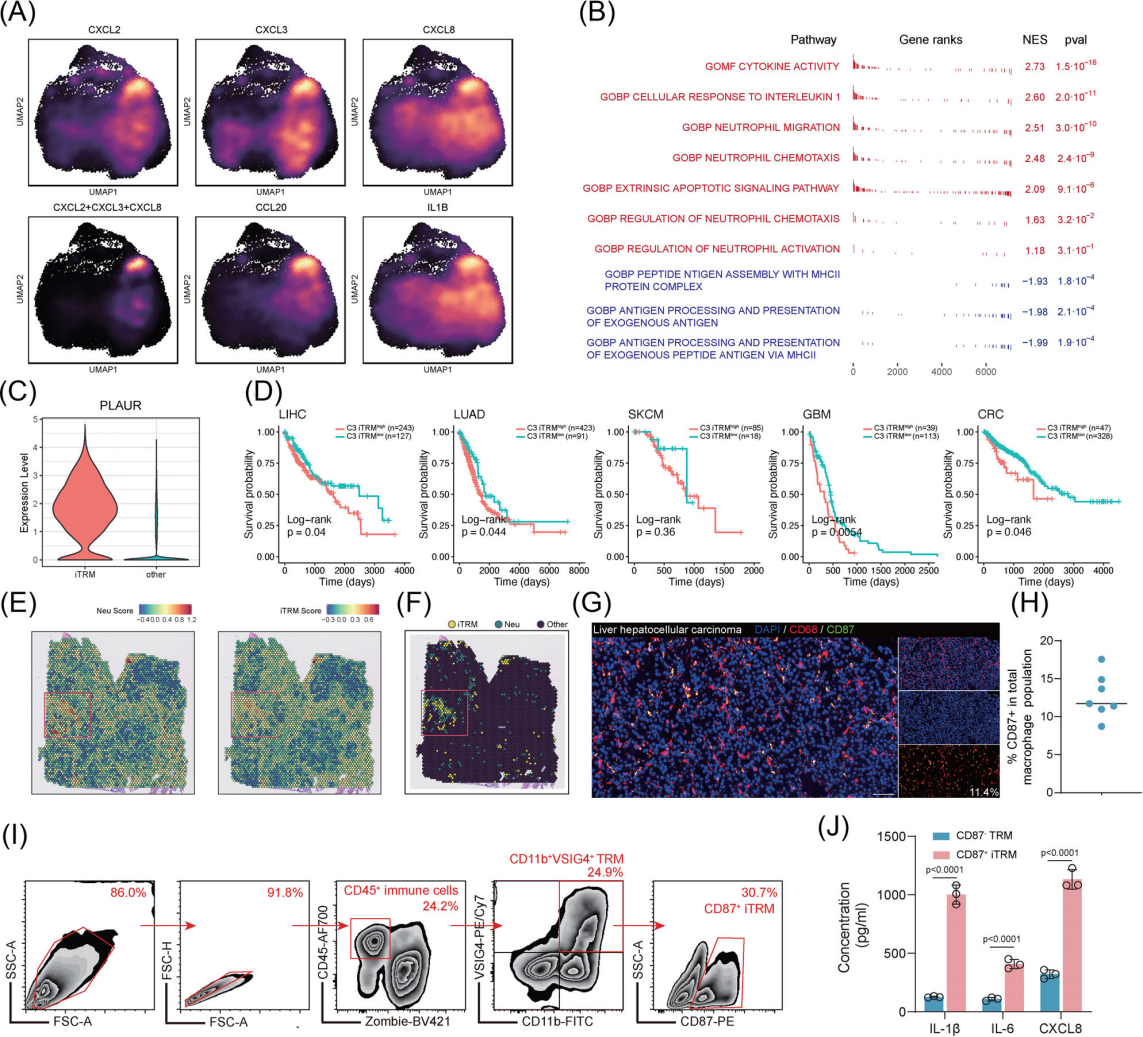
Identification of inflammatory tissue‐resident macrophages (iTRMs). (A) Density plots highlighting gene expression and co‐expression patterns of specific genes across individual cells. (B) Gene Set Enrichment Analysis (GSEA) showing enriched and depleted pathways in the C3_iTRM_IL1B subpopulation. (C) Violin plot demonstrating the specific expression of PLAUR in the iTRM subset within macrophages. (D) Kaplan–Meier survival curves comparing high versus low C3_iTRM_IL1B signature expression across cancer types (*p* < .05 indicates prognostic significance). (E) Spatial expression patterns of the C3_iTRM_IL1B signature and neutrophil signature in LUAD (lung adenocarcinoma), visualised as spatial spots with colour gradients from blue (low expression) to red (high expression). (F) Spatial co‐localisation of C3_iTRM_IL1B and neutrophils in LUAD revealed by spot deconvolution. (G) Representative images of DAPI, CD68 and CD87 in situ immunofluorescent staining in HCC samples (*n* = 7). Scale bars, 100 µm. (H) Quantification of the proportion of CD87+ TRM analysed by immunofluorescent staining and HALO Image Analysis Software. The percentage of CD87+ TRM represents the areas of CD87 divided by the CD68 positive area (*n* = 7). (I) Representative flow cytometry plot of gating strategy to identify the CD87+ iTRM subset in HCC samples. (J) ELISA quantification of IL‐6, IL‐1β and CXCL8 concentrations in culture supernatants from CD87− TRMs and CD87+ iTRMs. *p* Value by Student's *t*‐test; experiment was repeated three times. A *p* value of less than .05 indicates a statistical difference. Error bar represent mean ± SEM.

To further explore the conserved evolutionary patterns of TRM‐TAM characteristics and functions across diverse tissues in the TME, we focused on TRM subpopulations that exhibited consistent enrichment across multiple tissues, thereby enabling balanced comparative analyses. Both the C3_iTRM_IL1B and C8_Hypoxia_TRM_TIMP1 subpopulations met these selection criteria. However, since the C8_Hypoxia_TRM_TIMP1 subset demonstrated a quiescent state with minimal functional gene signatures, we prioritised in‐depth examination of the C3_iTRM_IL1B for subsequent investigations. C3_iTRM_lL1B is present in both tumour and normal tissues but is significantly enriched in tumour tissues. An analysis of the differentially expressed genes (DEGs) in C3_iTRM_lL1B revealed high expression of various inflammatory factors (such as IL‐1B and IL‐6) as well as chemokines including CCL20, CXCL8, CXCL2 and CXCL3. Additionally, CD87 (PLAUR) expression levels were significantly elevated in C3_iTRM_lL1B and may be involved in inflammatory responses. Based on this, we preliminarily defined C3_iTRM_lL1B as inflam‐TRM (iTRM). Furthermore, by examining the co‐expression of CXCL8, CXCL2 and CXCL3, we identified a strong specificity for C3_iTRM_lL1B in recruiting neutrophils (Figure 3A). Additionally, Gene Set Enrichment Analysis (GSEA) of C3_iTRM_lL1B indicated a significant up‐regulation of pathways related to neutrophil migration and chemokine signalling, while the MHC II‐mediated antigen presentation pathways were down‐regulated (Figure 3B). To validate our findings, we collected bulk RNA sequencing data from the The Cancer Genome Atlas (TCGA) database, which included samples from liver HCC (TCGA‐LIHC, *n* = 370), LUAD (TCGA‐LUAD, *n* = 514), melanoma (TCGA‐SKCM, *n* = 103), GBM (TCGA‐GBM, *n* = 152) and CRC (TCGA‐CRC, *n* = 375), totalling 1514 samples. Using C3_iTRM_lL1B marker genes as signatures (log_2_FC > 1, *p* < .05), we evaluated the correlation between these markers and neutrophil infiltration (as assessed by xCell) in each cancer type. We observed that the C3_iTRM_lL1B signature score was highly positively correlated with neutrophil infiltration across the five cancer types (Figure S8A).

To validate the presence of C3_iTRM_lL1B and their co‐localisation with neutrophils, we analysed spatial transcriptomic data from the five cancer types. Transcriptomic analysis revealed co‐expression of neutrophil score and C3_iTRM_lL1B signature (Figures 3E and S6A). Next, we defined the cellular identity of each spot based on deconvolution results. Notably, C3_iTRM_lL1B and neutrophils exhibited strong spatial co‐localisation (Figures 3F and S7A). Correlation analyses further confirmed significant spatial associations between these two cell types (Figure S7B). Quantification of neutrophil proportions in C3_iTRM_lL1B neighbourhood revealed markedly higher levels than random background distributions (Figure S7C). Subsequent permutation testing of neutrophil proportions in iTRM spot neighbourhoods demonstrated significantly higher mean neutrophil proportions compared with randomly permuted distributions (Figure S7D), providing additional support for C3_iTRM_IL1B‐neutrophil co‐localisation. Furthermore, we quantified spatial autocorrelation across cancer types using global Moran's *I* statistics. Results showed an average Moran's *I* value of .78 for neutrophil distribution, statistically significant (*p* < .001), further confirming substantial autocorrelation between iTRMs and neutrophils (Figure S7E). Cross‐cancer analysis indicated that SKCM specimens exhibited the strongest spatial correlation between iTRMs and neutrophils (*R* = .49, *p* < .001; Figure S7F), accompanied by a distinct stratified distribution pattern. This suggests potential formation of specialised organisational structures between iTRMs and neutrophils within the SKCM tissue microenvironment.

To further experimentally validate the existence of C3_iTRM_lL1B, we analysed single‐cell transcriptomic data and identified CD87 (PLAUR) as specifically expressed in this subset compared with other macrophage populations (Figure 3C). CD87 encodes the cell surface receptor uPAR, which participates in cell migration, matrix remodelling and inflammatory responses within the microenvironment. These functions align with the core activities of C3_iTRM_lL1B. Furthermore, CD87 membrane expression is primarily restricted to macrophages (Figure S8B), suggesting its utility as a candidate surface marker for isolating this iTRM subset. Therefore, we performed immunofluorescence co‐staining for CD68 and CD87 on human HCC sections, revealing CD87+ macrophage/iTRM‐like cells (Figure 3G). We further quantified the proportion of CD87+ cells within the total macrophage compartment across patient samples (Figure 3H), supporting the presence of a definable CD87+ macrophage subset (iTRMs) within the HCC TME.

Subsequently, we collected fresh tumour tissue samples from HCC patients and employed flow cytometry (FACS) to isolate CD87− TRM and CD87+ iTRM populations from human HCC tissues (Figure 3I). To functionally validate iTRM as a distinct inflammatory macrophage subset, we measured cytokine levels in TRM culture supernatants by enzyme‐linked immunosorbent assay (ELISA). CD87+ iTRMs secreted significantly higher IL‐1β, IL‐6 and CXCL8 compared with CD87− TRMs (Figure 3J), supporting for the computationally inferred iTRM inflammatory program. In addition, we observed significantly higher secretion of CXCL8, CXCL2 and CXCL3 in CD87+ iTRM compared with CD87− TRM (Figure S9A), supporting that iTRM is enriched for neutrophil‐attracting chemokines production. CD87+ iTRMs significantly enhanced neutrophil migration compared with CD87− TRMs in Transwell co‐culture assays (Figure S9B). Within 2 h, iTRMs induced substantially greater neutrophil migration to the lower chamber than TRMs (*p* < .05; Figure S9D, left), as quantified by flow cytometry. This demonstrates that iTRMs possess an enhanced capacity for paracrine neutrophil recruitment. To determine whether the enhanced neutrophil recruitment is mediated by CXCL8/CXCL2/CXCL3, we individually silenced these chemokines in iTRM (two independent siRNAs per target) and repeated the migration assay (Figure S9C). Knockdown of CXCL2, CXCL3 or CXCL8 each significantly reduced neutrophil migration compared with control (Figure S9D, median), indicating that iTRM‐driven chemotaxis depends on this chemokine program. In addition, pharmacological blockade of neutrophil CXCR1/2 signalling using SX‐682 significantly attenuated migration induced by iTRM supernatant (Figure S9D, right), further confirming that the effect is mediated through canonical neutrophil chemokine receptors.

To further elucidate the regulatory mechanisms underlying the phenotype of these TRM‐TAM subsets, we employed pySCENIC to analyse the distinct transcriptional factor activities across each subpopulation. Our analysis revealed that transcriptional factor activity patterns strongly correlated with tissue origins, as TRM clusters derived from the same tissue exhibited highly similar transcriptional factor signatures (Figure S9E). Strikingly, the C3_iTRM_IL1B subpopulation demonstrated remarkably elevated STAT3 activity, suggesting its pivotal role in orchestrating the interaction between C3_iTRM_IL1B and neutrophils while maintaining their proinflammatory state. This finding was consistently supported by the significant enrichment of IL‐6/JAK–STAT3 signalling pathway in this specific subpopulation.

Overall, these results reflect the highly inflammatory state of C3_iTRM_lL1B and their active recruitment of neutrophils. Previous studies have suggested that IL‐1B may have detrimental effects on neutrophils,^103^ leading to adverse clinical outcomes, prompting us to speculate that C3_iTRM_lL1B recruit neutrophils and influence their function through inflammatory molecules such as IL‐1B, leading to poor prognosis.

To further confirm our hypothesis and assess the clinical value of TRM‐TAM subpopulations, including C3_iTRM_lL1B, across cancer types, we constructed molecular signature scores for each subpopulation in the TCGA pan‐cancer cohort based on single‐cell transcriptomic features and explored their prognostic significance through survival analysis. By dividing patients into high and low expression groups based on feature scores, we found that a high C3_iTRM_lL1B signature score was significantly associated with shorter overall survival (OS) (Figure 3D). Multivariate Cox regression analysis also identified the C3_iTRM_lL1B signature as an independent predictor of poor prognosis across multiple cancer types (Figure S8D). Moreover, in a multi‐cancer analysis employing a random‐effects model, the iTRM signature revealed a consistent pattern of increased risk across diverse cancer types (Figure S8C), suggesting that C3_iTRM_lL1B may indeed be a conserved marker of poor prognosis across cancer types.

### 2.4 TME‐driven inflammatory remodelling of TRM into pro‐tumourigenic iTRM

**FIGURE 4:**
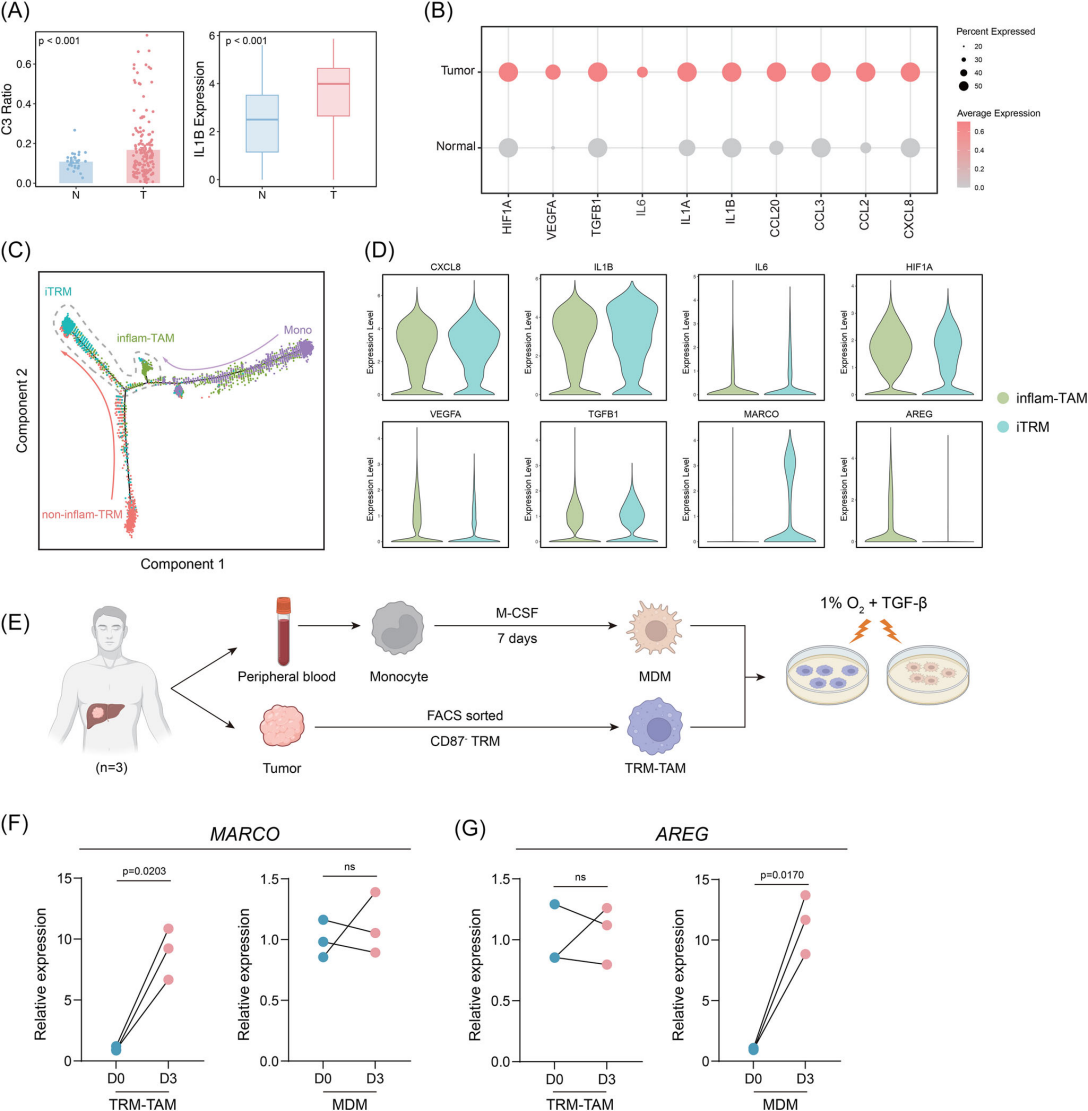
Tumour microenvironment‐driven inflammatory remodelling of TRM into pro‐tumourigenic inflam‐TRM (iTRM). (A) Left: proportional differences of C3_iTRM_IL1B between tumour and normal tissues (bar plot, mean ± SD, *p < .001, *t*‐test). Right: intergroup differences in IL‐1B expression within the C3_iTRM_IL1B subpopulation (box plot, median ± IQR, *p* < .01). (B) Bubble plot illustrating differential expression (tumour vs. normal) of chemokines, angiogenic factors and hypoxia‐responsive mediators in the C3_iTRM_IL1B subpopulation. (C) Pseudotime trajectory analysis depicting developmental paths of iTRM and inflam‐TAM, with distinct origins but convergent terminal inflammatory states. (D) Violin plots comparing shared inflammatory state genes and subpopulation‐specific functional genes between iTRM and inflam‐TAM. (E) Experimental scheme for culturing FACS‐sorted CD87− TRMs and M‐CSF‐differentiated MDMs from HCC patients (*n* = 3) under hypoxia (1% O_2_) with TGF‐β for 3 days. (F) qPCR analysis of MARCO and AREG expression in TRM‐TAMs and MDMs at the indicated time points (*n* = 3). qPCR was performed in technical triplicates and averaged per sample. *p* Value by paired‐Student's *t*‐test; a *p* value of less than .05 indicates a statistical difference. Error bar represent mean ± SEM unless otherwise indicated.

Interestingly, the proportion of C3_iTRM_lL1B in tumour samples was significantly higher than in normal samples (Figure 4A), and the expression of inflammatory molecules such as IL‐1B within C3_iTRM_lL1B also showed a similar trend, suggesting that the TME may specifically induce or remodel the C3_iTRM_IL1B, making it more conducive to tumour development.

To validate this hypothesis, we performed an in‐depth analysis of transcriptional differences between tumour‐derived and normal cells in C3_iTRM_lL1B, revealing significant up‐regulation of multiple functional molecules in tumour‐associated cells, including pro‐inflammatory factors (IL‐6, IL‐1B, IL‐1A, TGFB1), tissue‐remodelling mediators (HIF1A, VEGFA) and chemokines (CXCL8, CCL2) (Figure 4B). These expression signatures closely correlated with tumour malignancy. For example, the HIF1A–VEGFA axis may drive aberrant angiogenesis, whereas TGFB1 promotes metastasis through induction of EMT. Moreover, cross‐comparison between tumour‐specific genes highly expressed in C3_iTRM_IL1B and those up‐regulated across C3_iTRM_IL1B and other TRM‐TAM subpopulations revealed recurrent enrichment of IL‐1B, IL‐6 and CXCL8 in overlapping core gene sets (Figure S9I), suggesting that their elevated expression in C3_iTRM_IL1B may reflect both functional specialisation and microenvironmental constraints.

Notably, C3_iTRM_lL1B closely resembled known Mono‐derived inflammatory TAMs (inflam‐TAM) in both phenotypic characteristics and marker gene expression (Figure S9J), reinforcing its inflammatory state and motivating deeper exploration of its functional link to inflam‐TAM.^36^ Pseudotime trajectory analysis constructed by Monocle2^104^ showed that although TRM‐TAM (represented by C3_iTRM_lL1B) and Mono‐TAM originated from different developmental paths, both eventually converged t into a highly similar inflammatory terminal state (Figure 4C). Further analysis revealed that the C3_iTRM_lL1B and inflam‐TAM subtypes shared a conserved IL‐1B/IL‐6/CXCL8 signalling axis (Figure 4D). Particularly, both subtypes exhibited concurrent activation of hypoxia‐sensing pathways (e.g., HIF1A) and TGF‐β signalling within the TME. These observations suggest that microenvironmental pressures, such as hypoxia and TGF‐β exposure, may drive a convergent inflammatory phenotype in Mono‐TAM and TRM‐TAM. These findings further support additional justification for our classification of C3_iTRM_IL1B as iTRM. Interestingly, iTRM exhibited significantly higher expression of MARCO, while inflam‐TAM showed marked up‐regulation of AREG. This distinct receptor expression pattern suggests that iTRM and inflam‐TAM may maintain divergent functional adaptations to inflammatory signals, despite shared microenvironmental triggers. We next established an in vitro system using tumour tissue‐purified CD87− TRMs and peripheral blood MDMs. As outlined in the experimental scheme (Figure 4E), peripheral blood monocytes were differentiated with M‐CSF for 7 days to generate MDMs, while CD87− TRMs were FACS‐sorted from human HCC tumour tissues and used as the native TRM population prior to inflammatory exposure. Both populations were then exposed to 1% O_2_ plus TGF‐β to mimic tumour microenvironmental stress. These two macrophage lineages displayed distinct transcriptional responses: MARCO was significantly induced during ex vivo conditioning of CD87− TRMs (TRM‐TAM) but showed no comparable increase in MDMs, whereas AREG was preferentially induced in MDMs with minimal change in TRM‐TAMs (Figure 4F,G). These results further support that tissue‐resident TRM‐derived macrophages and MDMs retain divergent programs even under shared microenvironmental triggers.

Overall, C3_iTRM_lL1B exhibits unique pro‐tumour characteristics within the TME, and it shares a high degree of phenotypic convergence with peripheral‐derived inflam‐TAMs. This suggests that tumours may influence macrophages through ‘convergent evolution’, reshaping the inflammatory phenotypes of macrophages and making them key drivers of tumour malignancy, thereby creating an immune microenvironment more conducive to tumour survival and evasion, regardless of the developmental origins of the macrophages.

### 2.5 Dynamic transition of TRM from non‐inflammatory to inflammatory phenotypes in the TME

**FIGURE 5:**
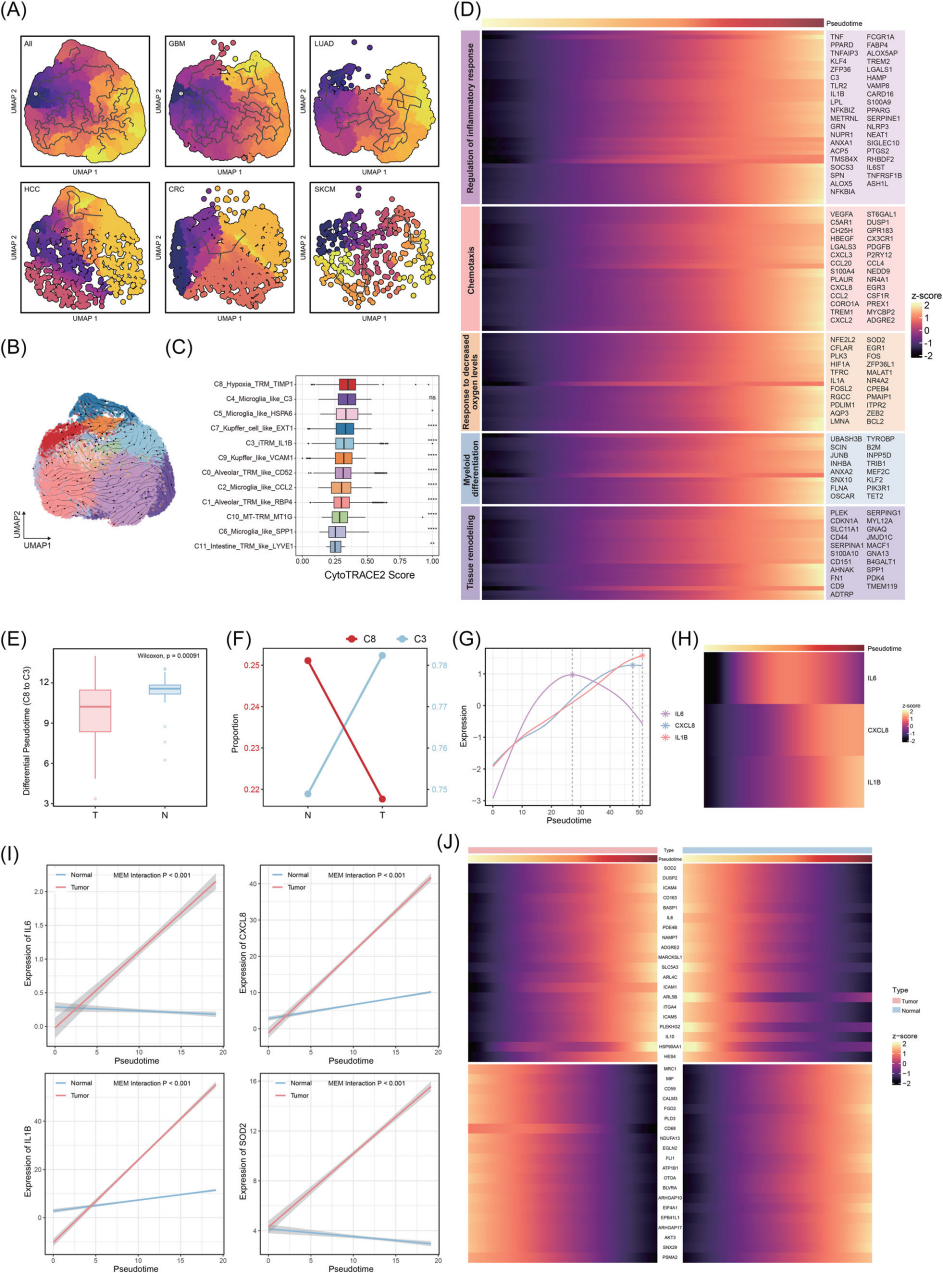
Dynamic transitions of inflammatory tissue‐resident macrophages (iTRM). (A) Pseudotime trajectory analysis (Monocle3) depicting global and tissue‐specific differentiation paths of TRM subpopulations. (B) RNA velocity vector field analysis illustrating differentiation directions of TRM subpopulations. (C) Box plot showing differentiation potential scores of TRM‐TAM subpopulations, with statistical comparisons by Wilcoxon rank‐sum test (*: *p* < .05, **: *p* < .01, ***: *p* < .001, ****: *p* < .0001). (D) Heatmap of gene expression dynamics along TRM lineage trajectories. (E) Box plot comparing evolutionary pseudotime required for transitions from C8 to C3 in tumour vs. normal samples. (F) Connected line plot contrasting proportional differences of C8 and C3 between tumour and normal samples. (G) Line plots showing expression trends of IL‐1B, CXCL8 and IL‐6 across TRM developmental pseudotime. (H) Heatmap of IL‐1B, CXCL8 and IL‐6 expression dynamics along TRM lineage trajectories. (I) Mixed‐effects model highlighting temporal expression divergence of selected genes in tumour vs. normal samples. (J) Heatmap of genes exhibiting opposing temporal expression trends in tumour versus normal samples.

The dynamic evolution of cells within the TME is a critical determinant of malignant progression. To elucidate the transition process of iTRM and the developmental trajectories of TRM‐TAMs, we performed pan‐cancer level pseudo‐time analysis and RNA velocity vector field modelling to reveal their differentiation patterns.^105^, ^106^ First, differentiation potential was calculated for each TRM‐TAM subset using CytoTRACE2.^107^ C8_Hypoxia_TRM_TIMP1 demonstrated the highest differentiation potential score and was consequently designated as the starting point for Monocle3^105^ pseudotime trajectory construction (Figure 5C). Subsequent pseudotime analysis revealed consistent positioning of the iTRM subset (C3_iTRM_lL1B) at the terminal trajectory endpoint, while the non‐iTRM subset (C8_Hypoxia_TRM_TIMP1) maintained stability at the trajectory origin (Figure 5A). Distinct evolutionary branch patterns were observed across different cancer types during this process. RNA velocity further validated this differentiation direction (Figure 5b), indicating that TRM‐TAMs undergo a conserved phenotype and functional transition from non‐iTRM to inflam‐TRM in tumours. Along this differentiation trajectory, TNF, IL‐1B and NFKBIZ were continuously activated (Figure 5D), while genes related to angiogenesis (VEGFA, PDGFB, HBEGF), matrix remodelling (FN1, CD44, SPP1) and hypoxia response (HIF1A, FOS, FOSL2) were gradually up‐regulated, marking the functional remodelling of TRM‐TAMs towards a pro‐tumourigenic inflammatory phenotype. Notably, the expression of chemokines CXCL8, CCL2, CXCL2 and CCL20 increased significantly with the differentiation process, suggesting their role in recruiting neutrophils, monocytes and Tregs, thereby exacerbating the immunosuppressive microenvironment.

Furthermore, the sequential up‐regulation of immunosuppressive factors such as TREM1, CSF1R and C5AR1 may consolidate the pro‐tumourigenic properties of terminal iTRM (Figure 5D). These dynamic changes collectively revealed the polarisation process of TRM‐TAMs from an early potential non‐inflammatory state to a terminal, highly inflammatory and immunosuppressive state. This process may drive tumour malignant progression through multiple mechanisms, including inflammation amplification, abnormal angiogenesis and matrix remodelling. The IL‐6/IL‐1B/CXCL8 regulatory axis, which plays a central role in iTRM, was also significantly enriched in the differentiation trajectory: the expression of IL‐1B and CXCL8 gradually increased with differentiation, while IL‐6 showed a characteristic of rapid saturation in the early stage (Figure 5G,H). These findings suggest that IL‐6 may initiate network activation by triggering JAK–STAT3‐mediated inflammatory cascades, potentially establishing a self‐reinforcing circuit with IL‐1B/CXCL8 to contribute to the stabilisation of the terminal inflammatory state in iTRM. This helps explain the dominant enrichment of the C3_iTRM_lL1B subpopulation in tumours and offers a potential theoretical basis for targeting key regulatory nodes (such as early blockade of IL‐6 signalling) as a strategy that could help mitigate the immunosuppressive state of the immune microenvironment.

It is noteworthy that previous studies have revealed a positive feedback regulatory network among IL‐6, IL‐1B and CXCL8.^63^, ^108^, ^109^ Thus, we perturbed IL‐6 signalling and examined downstream IL‐1β and CXCL8 outputs in CD87+ iTRMs. siRNA‐mediated knockdown of IL‐6 significantly reduced IL‐6 secretion and was accompanied by a marked decrease in IL‐1β and CXCL8 levels (Figure S9F, left). Consistently, functional blockade of IL‐6R similarly suppressed IL‐1β and CXCL8 production (Figure S9F, right). Moreover, pharmacological inhibition of STAT3 signalling with S3I‐201 attenuated IL‐1β and CXCL8 levels (Figure S9H), supporting an IL‐6–STAT3‐dependent amplification mechanism. Together, these results provide strong experimental evidence that CD87+ iTRMs sustain an inflammatory secretory program through an IL‐6–STAT3‐dependent amplification circuit that promotes IL‐1β and CXCL8 production. This self‐reinforcing inflammatory loop may constitute a core engine of tumour malignancy.

To further reveal the critical influence of the TME on the iTRM phenotype transition, we systematically compared the dynamic characteristics of non‐inflam‐TRM (niTRM) differentiation to iTRM in tumour and normal samples. Pseudo‐time analysis showed that the time required for cells in tumour samples to complete iTRM transformation was significantly shorter than that in normal tissues (Figure 5E), suggesting that the TME may accelerate the iTRM remodelling process through exogenous pressure. This conclusion is consistent with changes in the proportion of C3 and C8 subpopulations: in normal samples, niTRM (C8) accounted for a significantly higher proportion than in tumour samples, while iTRM (C3) was highly enriched in tumours (Figure 5F), indicating that tumours may shape pro‐tumourigenic TRM‐TAMs by selectively promoting the transformation of niTRM to iTRM.

Extending our investigation into the molecular driving factors of this process, we used a mixed effects model to identify genes that significantly contribute to the inflammatory remodelling of TRM (Figure 5H). Interestingly, the temporal expression trends of IL‐6, CXCL8 and IL‐1B were extremely different between tumour and normal samples. In tumours, the three were continuously and rapidly up‐regulated with the differentiation process, while in normal tissues, they showed a downward or slow upward trend (*p* < .001). Genes specifically up‐regulated in the tumour TRM differentiation trajectory included inflammatory factors (IL‐6, IL‐10), immunosuppressive receptors (CD163, ICAM1) and metabolic regulatory molecules (NAMPT, SLC5A3), while genes up‐regulated in the normal TRM trajectory involved homeostasis maintenance (MIF, CD68) and anti‐inflammatory functions (FLI1, EGLN2) (Figure 5I). These dynamic expression patterns suggested that the TME may have modulated the differentiation program of TRM‐TAM, dampening their typical anti‐inflammatory functions while promoting a pro‐tumourigenic signalling network centred on the IL‐6/IL‐1B/CXCL8 axis. This appeared to accelerate inflam‐TRM phenotype remodelling, potentially adapting TRM‐TAM into participants in malignant progression.

### 2.6 iTRM promoted tumour niche identification across cancer types

Our previous analysis had highlighted the crucial role of the iTRM phenotype in mediating inflammatory responses, recruiting immune cells and remodelling the stroma, suggesting its potential involvement in fostering a TME conducive to immune escape. To explore the clinical translational value of this phenotype, we integrated five TCGA pan‐cancer cohorts (*n* = 1514), which covered cancers including SKCM, CRC, LUAD, LIHC and GBM. We then conducted cross‐cancer TME classification research using the IOBR framework.^110^

**FIGURE 6:**
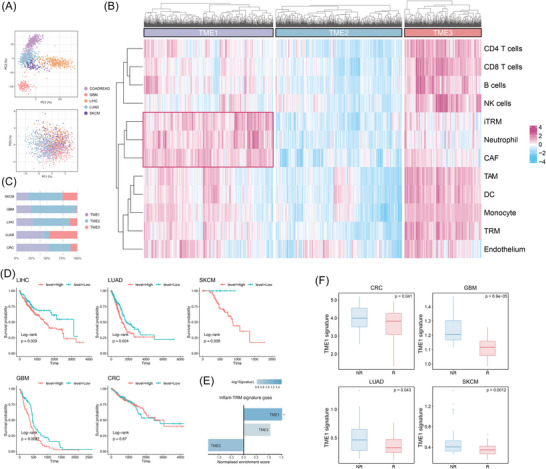
Identification of a pro‐tumourigenic niche mediated by inflammatory tissue‐resident macrophages (iTRM). (A) Principal component analysis (PCA) across TCGA tumour samples. Dots represent individual tumour samples, coloured by TCGA datasets (TCGA‐SKCM, TCGA‐CRC, TCGA‐LUAD, TCGA‐LIHC and TCGA‐GBM). (B) Bulk RNA‐seq deconvolution analysis using major cell‐type signatures identified from scRNA‐seq data based on ssGSEA (single‐sample Gene Set Enrichment Analysis) and tumour microenvironment (TME) classification. (C) Bar plot showing the distribution of three TME subtypes in TCGA cohorts. (D) Kaplan–Meier survival curves comparing overall survival between TME1 high‐score and low‐score subgroups across cancer types. (E) GSEA (Gene Set Enrichment Analysis) demonstrating iTRM signature enrichment in distinct TME subtypes. (F) Box plot comparing iTRM‐TME scores between immune checkpoint blockade (ICB)‐responsive and non‐responsive patients across cancer types.

Based on signatures of major cell types derived from single‐cell data, we performed bulk RNA‐seq deconvolution using single‐sample GSEA (ssGSEA). After correcting for tissue heterogeneity (Figure 6A), unsupervised clustering analysis was conducted. The optimal cluster number (*K* = 3) was determined by the elbow method (Figure S10A). This analysis led to the identification of three conserved TME subtypes across different cancer types: TME1 (iTRM‐specific type), TME2 (Immune‐Desert type) and TME3 (Immune‐Rich type) (Figure 6B). Each TME subtype showed a stable distribution pattern in various cancers (Figure 6C). Specifically, TME1 exhibited a unique pattern of coordinated infiltration of iTRM, neutrophils and cancer‐associated fibroblasts (CAFs) in the pan‐cancer context (Figure S10B). Meanwhile, the activities of granulocyte traffic and matrix remodelling pathways in TME1 were significantly higher than those in the other two subtypes (Figure S10C), indicating an actively inflamed and remodelled state. Whereas TME2 exhibited broad suppression of immune functions, TME3 demonstrated extensive activation across the immune system.

When comparing the gene signatures of each TME subtype, we found that TME1 highly up‐regulated chemokines such as CXCL1, CXCL2, CXCL5, CXCL8 and inflammatory cytokines like IL‐6 and IL‐1B (Figure S11A). These findings were highly consistent with the iTRM characteristics identified in single‐cell transcriptomics. TME3 showed significant specific expression of genes related to immune killing (GZMK), antigen presentation (HLA‐DQB2) and immune checkpoints (PDCD1). However, TME2 demonstrated down‐regulation of immune activation‐related genes. Further analysis revealed that the inflam‐TRM signature was significantly enriched in TME1, clearly distinguishing it from TME2 and TME3 (Figure 6E). Given this strong correlation, we defined TME1 as iTRM‐TME (iTRM‐specific TME).

To understand the molecular driving mechanisms of iTRM‐TME, we analysed the ligand–receptor interaction characteristics of each TME subtype. It was discovered that iTRM‐TME specifically activated the CXCL8–CXCR1/2 and CSF3–CSF3R signaling axes, which promoted neutrophil recruitment and expansion^111^ (Figure S11B). In addition, the combined actions of the CCL7–CCR1/2/3/5 and IL‐6–IL‐6ST pathways might create an Immunosuppressive microenvironment by recruiting Treg/myeloid‐derived suppressor cell (MDSC) and inducing PD‐L1 expression.^112^, ^113^ Meanwhile, the RSPO3–LGR4/LRP6 and ANXA1/SAA1–FPR2 signalling networks, by activating CAFs and the NF‐κB‐dependent inflammatory feedback loop, contributed to maintaining stromal sclerosis and the pro‐inflammatory phenotype of iTRM.^114^, ^115^

We then evaluated the impact of iTRM‐TME on patient survival. We constructed an iTRM‐TME score based on its characteristic gene and explored its prognostic significance in LIHC, LUAD, CRC, GBM and SKCM. Results demonstrated that patients with iTRM‐TME exhibited significantly shorter OS across malignancies except CRC (Figure 6D), indicating poor prognosis in these cancer types. Moreover, by analysing five RNA‐Seq cohorts (CRC, NSCLC, melanoma, GBM, *n* = 257) with immune checkpoint blockade (ICB) immunotherapy outcomes^116^, ^117^, ^118^, ^119^, ^120^ and one CRC metastasis cohort^121^ (GSE50760, *n* = 33), we found that the iTRM‐TME signature was highly associated with non‐response to ICB treatment in various cancers (Figure 6F) and significantly related to tumour metastasis in CRC (Figure S10D). These findings suggested that iTRM‐TME could serve as a potential biomarker for predicting prognosis and treatment resistance across different cancer types.

### 2.7 Prognostic and immunotherapeutic predictive value of TIR‐Sig

Next, we aimed to develop a pan‐cancer gene signature that precisely reflects TME features associated with iTRM and investigate its prognostic value. Building on previous findings, we incorporated characteristic genes of iTRM‐TME from bulk RNA‐seq data and iTRM‐specific markers from scRNA‐seq data into machine learning model construction. Our analysis utilised five TCGA cohorts (TCGA‐LIHC, TCGA‐GBM, TCGA‐CRC, TCGA‐LUAD, TCGA‐SKCM) and four additional RNA‐seq cohorts^122^, ^123^, ^124^, ^125^ (LIHC_te: ICGC‐LIRI‐JP, LUAD_te: GSE31210, CRC_te: GSE17536, GBM_te: GSE83300), with the analytical workflow illustrated in Figure S12A. These cohorts were divided into three groups: training set (TCGA_tr, *n* = 1049), validation set (TCGA_va, *n* = 446) and cancer‐specific test sets (LIHC_te, LUAD_te, CRC_te, GBM_te, *n* = 514).

**FIGURE 7:**
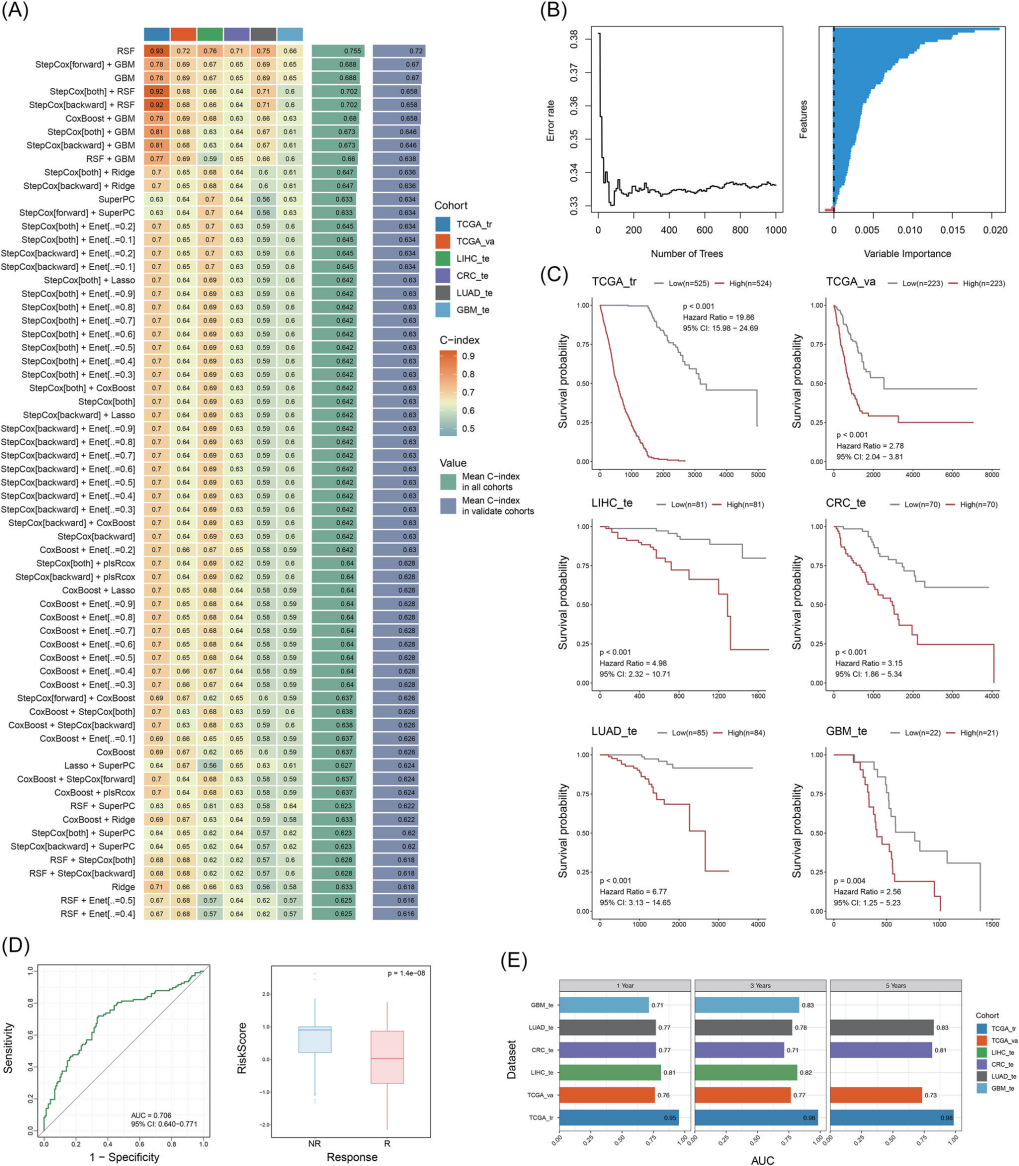
Prognostic and immunotherapeutic predictive value of the TRM inflammatory remodelling signature (TIR‐Sig). (A) A total of 101 prediction models were constructed using the Mime framework, and the *C*‐index of each model was calculated across all validation datasets. (B) Dynamic changes in prediction error (left) and distribution of variable importance (right) in the random survival forest (RSF) model. (C) Kaplan–Meier curves for overall survival (OS) based on the TIR‐Sig across multiple tumour cohorts (log‐rank test *p* values indicated). (D) Left: ROC curve of the TRM inflammatory remodelling signature (TIR‐Sig) for predicting response to immune checkpoint inhibitor (ICI) therapy in ICB‐treated cohorts; Right: Box plot comparing TIR‐Sig risk scores between ICI responders and non‐responders (*p* value shown). (E) Time‐dependent AUC values of the TIR‐Sig for predicting 1‐, 3‐ and 5‐year survival in training and validation cohorts.

In the training cohort, we employed an ensemble of 101 machine learning algorithms to develop predictive models. Using 10‐fold cross‐validation with five repeats (Figure S12B,C), we evaluated each algorithm's performance through mean *C*‐index calculations in validation and test sets. The random survival forest (RSF) model demonstrated optimal predictive accuracy with the highest mean validation *C*‐index (.72) (Figure 7A,B). The RSF model exhibited characteristic two‐phase prediction error dynamics: rapid optimisation during initial ensemble expansion (*n* < 200 trees) followed by progressive convergence (*n* ≥ 200 trees), consistent with Breiman's theoretical framework on random forest convergence (Figure 7B).^126^ We subsequently constructed the TIR‐Sig using the RSF model's internal variables.

Using median TIR‐Sig risk scores, patients were stratified into low‐risk and high‐risk groups across cohorts. High‐risk groups showed significantly reduced OS compared with low‐risk groups in the TCGA training data, validation dataset and cancer‐specific test sets (Figure 7C). Notably, TIR‐Sig effectively predicted ICB treatment response (area under the curve [AUC] = .706) in an additional independent immunotherapy cohort we collected (NSCLC: GSE126044, GSE135222; GBM: PRJNA482620; Melanoma: GSE91061; CRC: CodeOcean Capsule 8990462, *n* = 257), with higher risk scores significantly associated with ICB non‐response (Figure 7D), demonstrating its dual capability for pan‐cancer survival prediction and immunotherapy response evaluation. To comprehensively evaluate the predictive performance of TIR‐Sig across different immunotherapy regimens, we next performed subgroup analyses based on our existing immune checkpoint therapy cohorts supplemented with additional bulk RNA‐seq data from anti‐CTLA4 monotherapy patients (Figure S12D). The results demonstrate that TIR‐Sig maintained stable predictive capability in the anti‐PD1 monotherapy subgroup (*n* = 214, AUC = .65). In the anti‐CTLA4 monotherapy subgroup (*n* = 34), the observed AUC was .60, which is lower than that in the anti‐PD1 monotherapy subgroup. The anti‐PD1/CTLA4 combination subgroup (*n* = 32) achieved an AUC of .64, showing no significant difference compared with anti‐PD1 monotherapy. Notably, despite limited sample size, TIR‐Sig exhibited significantly enhanced predictive efficacy in the anti‐PD1/LAG3 combination subgroup (*n* = 11, AUC = .867). These findings suggest TIR‐Sig may possess superior clinical value in specific combination therapies, particularly anti‐PD1/LAG3 regimens.

Time‐dependent AUC analysis confirmed consistent predictive performance by TIR‐Sig across 1‐year, 3‐year and 5‐year survival endpoints, with all AUC values exceeding .7 in both training and validation sets (Figure 7E). Comparative evaluation against 95 published machine learning models revealed superior performance of TIR‐Sig, which ranked first in three datasets, second in two cohorts and third in one validation set (Figure S13A,B), thereby validating the clinical utility of this signature.

## 3 DISCUSSION

This study provides a comprehensive, cross‐tissue analysis of the tumour immune microenvironment, revealing a conserved pattern of inflammatory remodelling within the TRM compartment and its prognostic significance across cancers. Our findings establish iTRM as a distinct subset of TRM characterised by high expression of CXCL8, IL‐1B and IL‐6, potent neutrophil recruitment capabilities and significant associations with adverse clinical outcomes. These observations align with research highlighting the importance of macrophage polarisation in shaping the TME and influencing disease progression. A key finding of our study is the phenotypic convergence observed between TRMs and Mono‐TAMs within the TME. Despite their distinct developmental origins, both cell types appear to differentiate along a convergent pathway towards a pro‐inflammatory state, potentially indicating a shared response to tumour‐derived signals. This convergent evolutionary process appears driven by shared microenvironmental cues, such as hypoxia, inflammatory cytokines and matrix remodelling signals, which promote the activation of common signalling pathways and transcriptional programs. The study reveals that iTRM and inflam‐TAM share key signalling axes like IL‐1B/IL‐6/CXCL8, and both cell types show activation of hypoxia signalling (HIF1A) and TGF‐β pathways in the TME, suggesting the TME pressure contributes to the convergent inflammatory phenotype of TAMs and TRMs.

Previous studies have predominantly shown that TRMs typically exhibit an M2 phenotype, which is anti‐inflammatory and can be exploited by tumours to suppress the immune response, creating a conducive environment for tumour growth.^22^ However, our current research identifies an inflammatory subset of TRMs (iTRM) with an M1‐like pro‐inflammatory phenotype that exhibits an M1‐like pro‐inflammatory phenotype yet retains partial M2‐associated immunosuppressive function. As described in the previous study,^127^ TRMs can undergo significant inflammatory remodelling in the TME. The TME is a complex milieu filled with various factors such as hypoxia, inflammatory cytokines and abnormal ECM components. These factors can act as strong stimuli that override the normal regulatory mechanisms of TRMs. Hypoxia, for instance, is a common feature in tumours. It can activate specific signalling pathways within TRMs, leading to a shift in their phenotype. The activation of hypoxia‐inducible factors (HIFs) in TRMs, as a response to the hypoxic tumour environment, can up‐regulate genes associated with a pro‐inflammatory phenotype. In addition, continuous exposure to high levels of inflammatory cytokines like TNF‐α, which are abundant in the TME, can disrupt the normal polarisation balance of TRMs. Compared with the well‐studied pro‐tumour mechanisms of M2‐polarised TRMs and monocyte‐driven TAMs, such as promoting angiogenesis through the secretion of VEGF‐A, the pro‐tumour role of iTRM lies in its ability to recruit neutrophils and create an immunosuppressive microenvironment. Upon recruitment by iTRM, neutrophils may contribute to tumour growth by releasing NETs and other factors. This process may sustain a pro‐tumour inflammatory environment, potentially suppress anti‐tumour immune responses and could contribute to therapeutic resistance.

It is important to note that the traditional view of M1/M2 polarisation of macrophages is an oversimplification. Macrophages in the TME exhibit a high degree of plasticity and heterogeneity. They do not simply exist as two distinct polarised states but rather as a continuum of phenotypes.^128^ The TME is highly complex, with multiple factors acting in concert. Macrophages can respond to these diverse signals in a context‐dependent manner, resulting in phenotypes that cannot be neatly classified as either M1 or M2. For example, some macrophages may simultaneously express a combination of markers associated with the M1 and M2 phenotypes, or they may switch between different phenotypes during the progression of tumour development.^129^ Specifically, the transition from M1 to M2 typically resolves inflammation and promotes tissue remodelling, whereas the reverse transition triggers inflammatory responses. Moreover, tumours may exploit such plasticity to maintain a hybrid state, as exemplified by the iTRM phenotype. In this context, IL‐1β‐driven inflammatory cascades are amplified to recruit neutrophils (reflecting M1‐like attributes), whose NETosis releases proteases that activate fibroblasts. Meanwhile, CD206 and TREM2 sustain an immunosuppressive axis, which remodels the stroma and inhibits immune response (reflecting M2‐like functions). This integration of antagonistic polarisation axes transforms them into synergistic tools: IL‐1β maintains a pro‐tumourigenic microenvironment, which simultaneously requires M2‐like immunosuppression to evade immune attack. Furthermore, proteases released during NETosis may reprogram inhibitory receptors such as TREM2, thereby distorting polarisation dynamics and locking macrophages into a metastable state between polarisation extremes. Collectively, we define iTRMs as a distinct tumour‐corrupted M1/M2 hybrid state that transcends the classical M1/M2 dichotomy, while also exposing its limitations. Within the dynamic pathological ecosystem of the TME, macrophage phenotypes (particularly those of TRMs) likely represent not static binary choices but a continuous functional spectrum dynamically sculpted by local signalling networks. These states extend beyond the M1/M2 polarisation axis, instead unfolding across multidimensional axes of immunosuppression, stromal remodelling, angiogenesis and metabolic reprogramming to establish adaptive ‘phenotypic niches’ that accommodate tumour evolutionary pressures.

In addition, iTRM inflammatory remodelling may be regulated by upstream factors within the TME. Recent studies have highlighted the intratumoural microbiome as a critical determinant of immune phenotype shaping. Specifically, certain intratumoural microbes, such as Bifidobacterium and Lactobacillus species, can secrete metabolites like short‐chain fatty acids or directly interact with pattern recognition receptors (e.g., TLRs) on macrophages, inducing polarisation towards immunosuppressive phenotypes while also driving unique inflammatory phenotypes in subsets of macrophages.^130^, ^131^, ^132^ We hypothesise that the iTRM phenotype identified in our study may be partially driven by specific intratumoural microbial community structures; microbial metabolites could enhance the expression of inflammatory factors in iTRMs via activating signalling pathways such as NF‐κB, providing a novel perspective for understanding the origin and functional regulation of iTRMs.

Furthermore, interactions between iTRMs and other immune cell subsets in the TME appear to contribute significantly to the formation of immunosuppressive niches. While our study has confirmed that iTRMs recruit neutrophils via the CXCL8–CXCR1/2 axis, recent research suggests that iTRMs may form a synergistic regulatory network with Tregs and MDSCs.^133^, ^134^, ^135^, ^136^, ^137^ IL‐6 secreted by iTRMs can induce the proliferation and functional activation of Tregs via activating the JAK–STAT3 pathway, while simultaneously promoting the enrichment and retention of MDSCs within tumours. In turn, Tregs and MDSCs can maintain the inflammatory and immunosuppressive phenotype of iTRMs by secreting cytokines like TGF‐β, forming a positive feedback loop. Additionally, iTRMs may directly impair the cytotoxic function of CD8+ T cells through down‐regulation of MHC class II molecules and reduced antigen presentation. Concurrently, their secretion of chemokines such as CXCL2 could hinder effector T cell infiltration into tumour cores. Together with CAFs, iTRMs appear to contribute to both physical and immune barrier formation, suggesting this interactive network may collectively promote tumour immune escape.

The TME critically governs TRM‐TAM phenotypes and functions. Our data demonstrate that TME‐driven inflammatory remodelling of TRMs establishes a self‐reinforcing niche featuring granulocyte infiltration and ECM dysregulation. This process modulates TRM‐TAM differentiation programs, suppresses anti‐inflammatory functions and activates a pro‐tumourigenic IL‐6/IL‐1B/CXCL8 signalling axis, consistent with known links between matrix remodelling and TRM reprogramming.^138^ Furthermore, iTRM‐TME exhibits immune‐excluded characteristics with peripheral immune cell accumulation but inefficient core infiltration. Tumour‐derived factors likely generate physical–chemical barriers such as dense ECM networks restricting immune mobility, potentially exacerbated by NETs through DNA–protein scaffolds and protease‐mediated matrix remodelling. These collective mechanisms promote immune exclusion from tumour masses, ultimately attenuating anti‐tumour immunity.

The identification of the TIR‐Sig provides a valuable tool for predicting patient survival and response to immunotherapy. High TIR‐Sig scores are significantly associated with shorter OS and resistance to immunotherapy in independent cohorts, highlighting the clinical relevance of the inflam‐TRM phenotype. This signature may serve as a useful biomarker for stratifying patients and guiding treatment decisions. Notably, despite limited sample size, TIR‐Sig exhibited significantly enhanced predictive efficacy in the anti‐PD1/LAG3 combination subgroup. These findings suggest TIR‐Sig may possess superior clinical value in specific combination therapies, particularly anti‐PD1/LAG3 regimens. The differential predictive performance likely stems from distinct capacities of ICIs to modulate core inflammatory pathways defined by TIR‐Sig. Although the iTRM‐dominated immunosuppressive microenvironment characterised by TIR‐Sig is primarily driven by the IL‐6/IL‐1β/CXCL8 axis with neutrophil enrichment, iTRMs as TRMs intrinsically retain T cell suppressive functions. LAG3 inhibitors directly target macrophages by blocking LAG3–MHC‐II interactions, potentially more effectively disrupting immunosuppressive circuits, which explains the significantly enhanced predictive sensitivity in anti‐PD1/LAG3 therapy. In contrast, CTLA4 inhibitors primarily act during T cell priming in lymph nodes, likely exhibiting limited regulatory capacity over TRM‐driven inflammatory microenvironments, resulting in relatively constrained predictive efficacy. While PD1 inhibitors can partially influence macrophage phenotypes through mediators such as IFN‐γ, their predominantly indirect mechanisms may be insufficient to reverse the sustained inflammatory state mediated by iTRMs, thus demonstrating only moderate predictive efficacy in monotherapy or CTLA4 combination settings. These observations indicate that the clinical utility of TIR‐Sig as a biomarker depends on therapeutic strategies targeting specific myeloid pathways. We must emphasise, however, that given the predominance of anti‐PD1 monotherapy cases in current public cohorts, conclusions regarding anti‐CTLA4 monotherapy and combination subgroups require validation in expanded sample sets and should be interpreted cautiously.

Our study has several important implications for future research and clinical practice. First, it provides a strong rationale for targeting inflam‐TRM as a therapeutic strategy for improving cancer outcomes. Disruption of the IL‐6/IL‐1B/CXCL8 signalling axis or inhibition of neutrophil recruitment may represent promising approaches for reversing the pro‐tumourigenic activities of these cells. Given the study's findings that the TME modulates TRM‐TAM differentiation programs, inhibiting normal anti‐inflammatory functions and activating a pro‐tumourigenic signalling network, therapeutic strategies could also focus on restoring normal TRM‐TAM function. Second, our findings highlight the importance of considering the interplay between TRMs and Mono‐TAMs in the TME. Combination therapies that target both cell types may be more effective than strategies that focus on either population alone. In the context of liver metastasis, simultaneously blocking monocyte recruitment and macrophage proliferation could effectively shift the hepatic microenvironment from an immunosuppressive to an immunostimulatory state, representing a potential therapeutic avenue. Finally, the TIR‐Sig provides a valuable tool for identifying patients who are most likely to benefit from therapies targeting the TME. The TIR‐Sig signature also shows a high correlation with ICI treatment response rates, further supporting its potential clinical utility. The high correlation between the TIR‐Sig and ICI treatment response rates further validates its potential clinical utility.

In conclusion, this study identifies a conserved inflammatory remodelling program that transforms TRMs into iTRM, a distinct pro‐tumourigenic subset. These cells exhibit M1‐like pro‐inflammatory characteristics with high expression of CXCL8, IL‐1B and IL‐6, driving neutrophil recruitment, ECM dysregulation and multi‐cancer immunosuppression. Our findings reveal phenotypic convergence between TRMs and Mono‐TAMs under TME cues including hypoxia and inflammatory cytokines, demonstrating shared evolution towards pro‐inflammatory states through common signalling axes. The TIR‐Sig correlates with poor OS, immunotherapy resistance and immune‐excluded TME1 subtypes, challenging the M1/M2 paradigm by reframing macrophage plasticity as a continuum. Collectively, these insights redefine TRMs as dynamic drivers of TME co‐evolution, providing a mechanistic framework to target iTRM networks via neutrophil recruitment blockade or anti‐inflammatory function restoration, while advancing TIR‐Sig as a subtype‐specific biomarker for personalised cancer therapy.

This study has several limitations. First, while our experimental and analytical approaches confirm the existence and inflammatory phenotype of iTRMs, the majority of findings rely on computational inference. The precise regulatory mechanisms require further elucidation, with therapeutic targets for modulating or reversing the iTRM phenotype remaining to be explored. Second, the specific regulatory effects of iTRMs on neutrophils following recruitment remain unclear. Third, although our analyses indicate the potential clinical utility of TIR‐Sig, the reliability of these findings necessitates validation through large‐scale cohorts and in vivo experiments.

## 4 METHODS

### 4.1 scRNA‐Seq data download and pre‐processing

Single‐cell RNA sequencing data from patients with LUAD (GSE131907, GSE134355, E_MTAB_6149), HCC (PRJCA007744), cutaneous melanoma (GSE189889, GSE215121), GBM multiforme (GSE162631, GSE163120, GSE173278) and CRC (GSE178341) were acquired from Gene Expression Omnibus (GEO), GSA and BioStudies databases. Raw gene expression matrices from each study were processed using the Scanpy^139^ package. Quality control filtering retained cells with mitochondrial gene content <15% and gene counts between 200 and 8000. Subsequently, doublet removal was performed using the Scrublet^140^ package, which calculates a doublet probability score per cell, automatically determines discrimination thresholds through bimodal analysis of score distributions and excludes all identified doublets to prevent analytical bias.

### 4.2 Preprocessing and clustering single‐cells

We standardised the raw gene expression data using Scanpy's sc.pp.normalize_total() and sc.pp.log1p() methods, screening highly variable genes for dimensionality reduction. Principal component analysis (PCA, n_pcs = 30) was performed followed by data integration across samples using the Harmony algorithm with a maximum of 50 iterations. Based on the Harmony‐corrected embedding matrix, we constructed a *k*‐nearest neighbour graph (n_neighbours = 10, n_pcs = 30) and performed Leiden clustering (resolution = .5) with UMAP visualisation. This process effectively eliminated inter‐sample batch effects while preserving biologically relevant heterogeneity, providing a robust basis for subsequent cell subpopulation identification. For cell type annotation, DEGs in each Leiden cluster were calculated via sc.tl.rank_genes_groups() (*p* < .05, log_2_FC > 1), and canonical marker gene expression patterns were visualised using sc.pl.dotplot(). Major lineages were annotated based on established markers. The preprocessing pipeline, comprising normalisation, dimensionality reduction, Harmony integration and differential gene analysis, was subsequently reapplied to each major lineage to resolve subtype identities.

### 4.3 Cell enrichment analysis

For each TRM cell subset, we evaluated its distribution pattern in different tissues by calculating the aforementioned OR values.^141^

### 4.4 Identification of TRM gene signatures

As described in the previous study,^142^ this study used T cell attraction, T cell inhibition, M1 and M2 myeloid cell feature sets to calculate gene set scores in each TRM cell subset to evaluate the key functions of TRM.

### 4.5 Pathway analysis

GSEA enrichment analysis was performed using the R package clusterProfiler based on gene expression matrix. The related functional gene sets were downloaded from the MSigDB database.

### 4.6 Cell trajectory

To delineate the differentiation trajectory of TRM subsets and infer pseudotime ordering, we performed single‐cell trajectory analysis using Monocle3. Differentiation trajectories were reconstructed via the learn_graph() function, with pseudotime values computed using order_cells(). Spatiotemporally dynamic genes showing significant trajectory‐associated changes (*q* < .01) were identified through graph_test(). Additionally, transcriptional vector fields of TRM subsets were inferred using scTour to predict developmental pseudotime and kinetic properties. Differentiation potential was quantified for each TRM subset using CytoTRACE2.

### 4.7 Transcription factor analysis

A fast python‐based implementation of single‐cell regulatory network inference and clustering (pySCENIC, version 0.12.1) was used to infer the transcription factors regulating each TRM‐TAM subsets. The pySCENIC computational process mainly consists of three parts. The first part is to use the co‐expression between genes to infer candidate regulatory modules. The algorithm used is the GRNBoost2 algorithm, which is based on the random forest model and can quickly identify co‐expression modules. The second part is to use the motif enrichment method to improve the co‐expression module, mainly using the cisTarger module method in the pySCENIC computational process. The third part is to use the gene set scoring algorithm AUCell to score the activity of transcription factors and their possible downstream target genes (called regulons), so as to find the transcriptional regulation patterns of different cell groups.

### 4.8 Bulk‐RNA‐seq data download and processing

RNA‐Seq data for LUAD (TCGA‐LUAD), cutaneous melanoma (TCGA‐SKCM), GBM (TCGA‐GBM), HCC (TCGA‐LIHC) and CRC (TCGA‐CRC) were acquired from the UCSC XENA platform (https://xena.ucsc.edu/), with pre‐processed datasets incorporating batch correction and gene annotation directly utilised for subsequent analyses. This yielded a combined cohort of 1514 tumour samples curated from TCGA and GEO repositories. Additional validation cohorts comprised an ICI cohort (*n* = 257) integrating data from GSE126044 (NSCLC), GSE135222 (NSCLC), PRJNA482620 (GBM), GSE91061 (Melanoma) and CodeOcean Capsule 8990462 (CRC); a CRC metastasis cohort (GSE50760; *n* = 33); and a machine learning test set (*n* = 514) incorporating ICGC‐LIRI‐JP (LIHC_te), GSE31210 (LUAD_te), GSE17536 (CRC_te) and GSE83300 (GBM_te) datasets.

### 4.9 Survival analysis

To evaluate the clinical prognostic utility of genes characterising TRM cell subpopulations, we performed survival analysis using the survival (v3.7.0) and survminer (v0.4.9) packages in R. Kaplan–Meier methodology generated survival curves with OS as the primary endpoint. Between‐group differences were assessed by log‐rank tests (significance threshold: two‐sided *p* < .05), while Cox proportional hazards models quantified hazard ratios (HR) with 95% confidence intervals. Within the random‐effects model framework, we use the restricted maximum likelihood method to assess heterogeneity among different cancer types.

### 4.10 Immune infiltration analysis

We employed the xCell^143^ algorithm to estimate immune cell infiltration levels from bulk RNA‐seq samples, enabling robust evaluation of the correlation between TRM cells and neutrophil infiltration.

### 4.11 Spatial‐RNA‐seq data download and processing

Spatial transcriptomic data of lung cancer (LUAD), skin melanoma (SKCM), GBM, HCC (LIHC) and CRC involved in this study were obtained from the SCAR Open Platform (http://www.scaratlas.com/).^144^ The pre‐processed data (including batch correction and gene annotation) provided by the platform were subsequently analysed and visualised by the Seurat^145^ package.

### 4.12 Spatial transcriptomics deconvolution

To resolve cellular heterogeneity in spatial transcriptomics data, we performed deconvolution analysis using SPOTlight (v1.6.7) implemented in R. Cell‐type proportions were estimated by integrating matched single‐cell RNA‐seq signature as signature matrices. Spatial distributions of cell populations were visualised using spot‐level abundance scores. Neighbourhood analysis was performed using *k*‐nearest neighbours to define localised cellular interactions within the spatial transcriptomic architecture. For each iTRM spot, we defined its local neighbourhood as the *k*‐nearest neighbours in physical space. The spatial co‐localisation enrichment ratio *E* was calculated for *k* values ranging from 5 to 50 in increments of 5. The enrichment ratio *E* is calculated as the ratio of the observed mean proportion of neutrophils within iTRM neighbourhoods to the expected mean proportion derived from 1000 random spatial permutations. Detailed computational methodology is provided in the spatial permutation test description section. We then computed the absolute difference in enrichment ratio between consecutive k values, Δ*E* = |*E*(*ki*) − *E*(*ki*_−__1_)|, identifying the start of the stable *k* interval as the first point where Δ*E* < .05, and its end where Δ*E* subsequently exceeded .05. Within this stable *k* interval, the *k* value corresponding to the peak enrichment ratio was selected as the final parameter for neighbourhood definition. This dual‐criterion approach ensures the capture of biological signal while mitigating noise from undersampled neighbourhoods (small *k*) or signal dilution from excessively large neighbourhoods (large *k*).

### 4.13 Spatial permutation test

To statistically demonstrate the spatial association between iTRM and neutrophils, we employed a permutation test. We formulated the null hypothesis positing spatial independence between iTRM and neutrophils, wherein the observed neutrophil proportions within neighbourhoods would arise solely through random distribution. We then performed random permutations of neutrophil proportion values across all spots (preserving the original data distribution while disrupting spatial correlations). Following each permutation, we recalculated the mean neutrophil proportion within iTRM spot neighbourhoods. This procedure was iterated 1000 times (n_perm = 1000) to generate a null distribution. We subsequently computed the permutation distribution mean (perm_mean), enrichment ratio (*E*) and permutation test *p* value. Here, the enrichment ratio is defined as the ratio of the mean neutrophil proportion within iTRM neighbourhoods before permutation to that after permutation.

### 4.14 Spatial autocorrelation control

Our permutation strategy inherently preserves the spatial autocorrelation structure because we only randomly permute cell labels while retaining their spatial coordinates. This generates a null distribution that accounts for spatial structure, thereby ensuring valid inference. Additionally, we quantified spatial autocorrelation across different cancer types using the global Moran's *I* statistic.

### 4.15 Cell isolation and polarisation of macrophages

Human peripheral blood mononuclear cells (PBMCs) were isolated from fresh blood by density gradient centrifugation with Ficoll‐Paque Plus (Sigma). Human monocytes were purified from PBMC using the EasySep™ Human Monocyte Isolation Kit (Stemcell Technologies) according to the manufacturer's protocol. For in vitro differentiation of monocytes into human macrophages, isolated monocytes were cultured in complete RMPI1640 supplemented with 10% FBS (Gibco) in the presence of 20 ng/mL recombinant human M‐CSF (Peprotech) for 7 days.

### 4.16 TRM isolation and culture

Primary TRMs were isolated from human liver HCC samples obtained from surgery. Every sample was washed with PBS and cut into small pieces (<1 mm^3^), transferred into 5 mL 1640 medium containing collagenase IV (1 µg/mL) (ThermoFisher Scientific) and subsequently incubated for 30 min on a 37°C shaker. Subsequently, 4 mL PBS was added to dilute the suspension, and then a 70‐µm cell mesh was used to filter the suspension. After centrifugation at 100 × *g* for 5 min, we collected the cell pellet for follow experiment or resuspended it with cell preservation liquid. Single‐cell suspension was incubated with indicated antibodies in PBS containing 1% foetal bovine serum for 30 min at 4°C. After washing with PBS, the cells were resuspended in PBS and sorted by flow cytometry. TRMs were cultured in RPMI1640 with 10% FBS respectively.

In some experiments, 4 × 10^5^ TRMs were cultured alone for 48 h, and the supernatants were collected for ELISA quantification of CXCL2, CXCL3, CXCL8, IL‐1β and IL‐6.

In some experiments, CD87+ iTRMs were transfected with IL‐6 siRNAs for knockdown or treated with IL‐6Rα antibody (R&D Systems) or STAT3 inhibitor S3I‐201 (25 µM) (Sigma–Aldrich) prior to ELISA analysis.

### 4.17 Isolation of human neutrophils from peripheral blood

Neutrophils were isolated freshly from whole blood of healthy donors, processed anonymously, using a human neutrophil isolation kit as per the manufacturer's instructions (STEMCELL Technologies).

### 4.18 Neutrophil migration assay in a transwell system

FACS‐sorted CD87− TRMs or CD87+ iTRMs (1.5 × 10^5^ cells per well) were seeded in the lower wells in 600 µL RPMI‐1640 containing .1% FBS and cultured for 24 h. Peripheral blood neutrophils were resuspended in RPMI‐1640 containing .1% FBS (1 × 10^6^ cells/mL), and 100 µL (1 × 10^5^ cells) was then added to the upper inserts of a Transwell chamber with an uncoated 3‐µm pore polyester membrane. After 2 h, cells in the lower chamber were collected and migrated neutrophils were quantified by flow cytometry. In some experiments, CD87+ iTRMs were transfected with scrambled control siRNA or two independent siRNAs targeting CXCL2, CXCL3 or CXCL8 and cultured for 24 h prior to the migration assay. Where indicated, neutrophils were treated with SX‐682 (1 µM) or DMSO before seeding.

### 4.19 Enzyme‐linked immunosorbent assay

Concentrations of the inflammatory cytokines and chemokines in the supernatants from ex vivo culture systems were detected using ELISA kits according to the manufacturer's instructions (R&D Systems, Abcam).

### 4.20 Hypoxia/TGF‐β stress exposure in macrophages

FACS‐sorted CD87− TRMs and peripheral blood MDMs were generated as described in *Methods* section (Section 4.15 and 4.16), respectively. Cells were then subjected to tumour‐like stress exposure under hypoxic conditions (1% O_2_) in the presence of TGFβ1 (10 ng/mL; PeproTech) for 72 h. Macrophages were harvested for downstream analyses.

### 4.21 Flow cytometry

For cell surface marker analysis, cells were resuspended in PBS containing 1% FBS and stained with fluorescent‐conjugated antibodies against CD45, CD11b, VSIG4 and CD87 for 30 min at 4°C. All analyses were conducted by cytoFLEX LX.

### 4.22 Immunofluorescence

Paraffin‐embedded samples were sectioned at 3 µm thickness. A pressure cooker performed antigen retrieval for 15–20 min in .01 M citrate buffer (pH 6.0). Then, sections were blocked in PBS containing 10% donkey serum or 2% bovine serum albumin for 1 h at room temperature. For immunofluorescence, by using a PANO 7‐plex kit (PANOVUE) according to the manufacturer's instructions, samples were incubated with dilutional primary antibodies against CD68 and CD87 (1:200), DAPI was then used to counter the nuclei. All images obtained by laser scanning confocal microscopy (LSM880, Zeiss or Vectra Polaris) and using HALO Image Analysis Software or Imaris 9.0 Microscopy Image Analysis Software for further analysis.

### 4.23 RT‐qPCR

RNA was extracted from indicated cells, and reverse transcription of the first‐strand cDNA was performed using a reverse‐transcription kit (Promega). The qRT‐PCR assay was conducted on the Bio‐Rad SPX (96 or 384) system with a 2X SYBR Green mix (Life, Carlsbad, CA, USA). The data were normalised to the expression of GAPDH.

### 4.24 Identification and characterisation of iTRM‐rich TME

TME characterisation was performed using the IOBR bioinformatics framework.^110^ Primary tumour samples from TCGA‐LUAD, TCGA‐GBM, TCGA‐LIHC, TCGA‐SKCM and TCGA‐CRC datasets were included. Normalisation corrected tissue heterogeneity, with PCA validating correction efficacy. Following normalisation, ssGSEA quantified immune function scores. *Z*‐score normalised data underwent unsupervised clustering via IOBR's tme_cluster() function to identify conserved TME subtypes across cancer types. Differential gene expression analysis for each TME subtype employed Seurat's FindMarkers function. Finally, subtype‐specific ligand–receptor interactions were calculated using the LR_cal() function.

### 4.25 Machine learning‐based prognostic modelling

The TIR‐Sig was developed using the Mime^146^ software package. We analysed eight independent cohorts spanning five cancer types (LIHC, GBM, CRC, LUAD, SKCM), with 70% of TCGA‐LIHC, TCGA‐GBM, TCGA‐CRC, TCGA‐LUAD and TCGA‐SKCM samples constituting the training set and 30% the validation set. Cancer‐specific test sets comprised ICGC‐LIRI‐JP, GSE31210, GSE17536 and GSE83300. Through systematic integration of 15 base algorithms (including RSF, CoxPH and gradient boosting machine), eight feature selection methods (e.g., minimum redundancy maximum relevance, stability selection) and seven ensemble strategies (weighted voting, stacked generalisation, Bayesian model averaging), we explored 101 machine learning configurations. The ML.Dev.Prog.Sig() function implemented parallelised grid search for hyperparameter optimisation while computing bootstrap‐corrected Harrell's *C*‐index. Time‐dependent AUC (1/3/5‐year survival landmarks) was evaluated via cal_AUC_ml_res(), and head‐to‐head comparisons against published models were conducted using cal_cindex_pre.prog.sig() through log‐rank testing and decision curve analysis. All analyses were performed in R 4.3.1 following TRIPOD guidelines.

This study employed a rigorous 10‐fold cross‐validation with five repeats (totalling 50 independent iterations) to evaluate the stability of the RSF model. Using the createFolds function, we performed stratified sampling on the training set samples across five repetitions, each time partitioning the data into 10 subsets preserving the original event ratio. while the remaining nine subsets formed the training set. The RSF model was then constructed using the rfsrc function (key parameters: ntree = 1000, nodesize = 5, splitrule = ‘logrank’). After model training, the *C*‐index on both the current training set and test set was concurrently assessed using the concordance.index function, and the results from all 50 iterations were fully recorded. Based on this complete dataset, key statistical metrics (mean, standard deviation, interquartile range, confidence intervals) were calculated. Visualisation plots were generated to display the evolution trajectory of the *C*‐index across iterations for both the training and test sets, including mean reference lines, providing a comprehensive assessment of model stability and generalisability. Bootstrap resampling with 1000 iterations was further used to assess model stability. In each iteration, a new sample of the same size as the original training set was drawn with replacement. The model was trained on this bootstrap sample and its performance was validated on the original training set. Finally, the coefficient of variation and 95% confidence interval for the *C*‐index were calculated.

### 4.26 Predictive performance validation in the ICB cohort

ICB treatment response prediction analysis was performed using the pROC software package (v1.18.4). Binary response labels were coded as responder = 1 (complete/partial remission) and non‐responder = 0 (stable/progressing disease) according to RECIST v1.1 criteria. Subject operating characteristic (ROC) curves were generated by logistic regression modelling of standardised risk scores versus dichotomous response outcomes, and the AUC was calculated using the nonparametric trapezoidal method with 95% confidence intervals estimated by 2000 Bootstrap resampling. Statistical significance of predictive performance was assessed by comparing random classifications (AUC = .5) via DeLong test.

### 4.27 Statistics

Python and *R* were used for data analysis and visualisation. Comparisons between groups were made using the Student's *t*‐test. Survival analysis was performed using the Kaplan–Meier method and comparisons were made using the log‐rank test. Correlation analysis was performed using Spearman correlation coefficients. Statistical methods and sample sizes (*n*) are provided in the figure legends unless otherwise stated. *p* Values < .05 were considered statistically significant, and significance levels denoted as *p < .05, **p < .01, ***p < .001, ****p < .0001 and ns (*p* > .05) denoted no significant difference.

## AUTHOR CONTRIBUTIONS

WK. W., JH. Y., X. J., GB. L., X. Y. and ZK. M. were responsible for the conceptualisation and design of the study. Data collection was carried out by WK. W., ZH. H., PX. Z., ZL. H., YH. Z. and C. W. Data analysis was conducted by WK. W., ZH. H., JZ. L., ZR. L. and BB. G. The experimental design and validation were completed by WK. W., ZB. C., YH. H. and MZ. K. Manuscript writing and contribution were completed by WK. W., X. C., PX. Z., Y. Z., XY. Z. and YF. Y. All authors made contributions to the article and approved the submitted version.

## CONFLICTS OF INTEREST STATEMENT

The authors declare no conflicts of interest.

## CODE AVAILABILITY

This study utilised publicly available packages with R version 4.3.3. All software and algorithms used in this study are publicly available and listed in the *Methods* section. All code generated for analysis is available from the authors upon request.

## ETHICS STATEMENT

Samples were collected from Sun Yat‐sen University Cancer Center, Sun Yat‐sen University, Guangzhou, China. The informed consent was obtained from all subjects in the human studies.

## Supporting information



Supporting infromation

Supporting infromation

## Data Availability

The datasets utilised in this study are publicly available as detailed in the Supporting Information. Additional data requests may be directed to the corresponding author.
