# Supplementary material for: Integrative cross‐tissue and spatially resolved single‐cell profiling uncovers tumour‐educated inflammatory remodelling of tissue‐resident macrophage ecosystem with immunotherapeutic prognostic significance in pan‐cancer
Source: Clin Transl Med. 2026 Feb 8;16(2):e70608. doi: 10.1002/ctm2.70608 (PMC12883038; doi:10.1002/ctm2.70608)
Supplement: Supplementary file 1 — Supporting infromation [file CTM2-16-e70608-s002.pdf]

Fig.S1

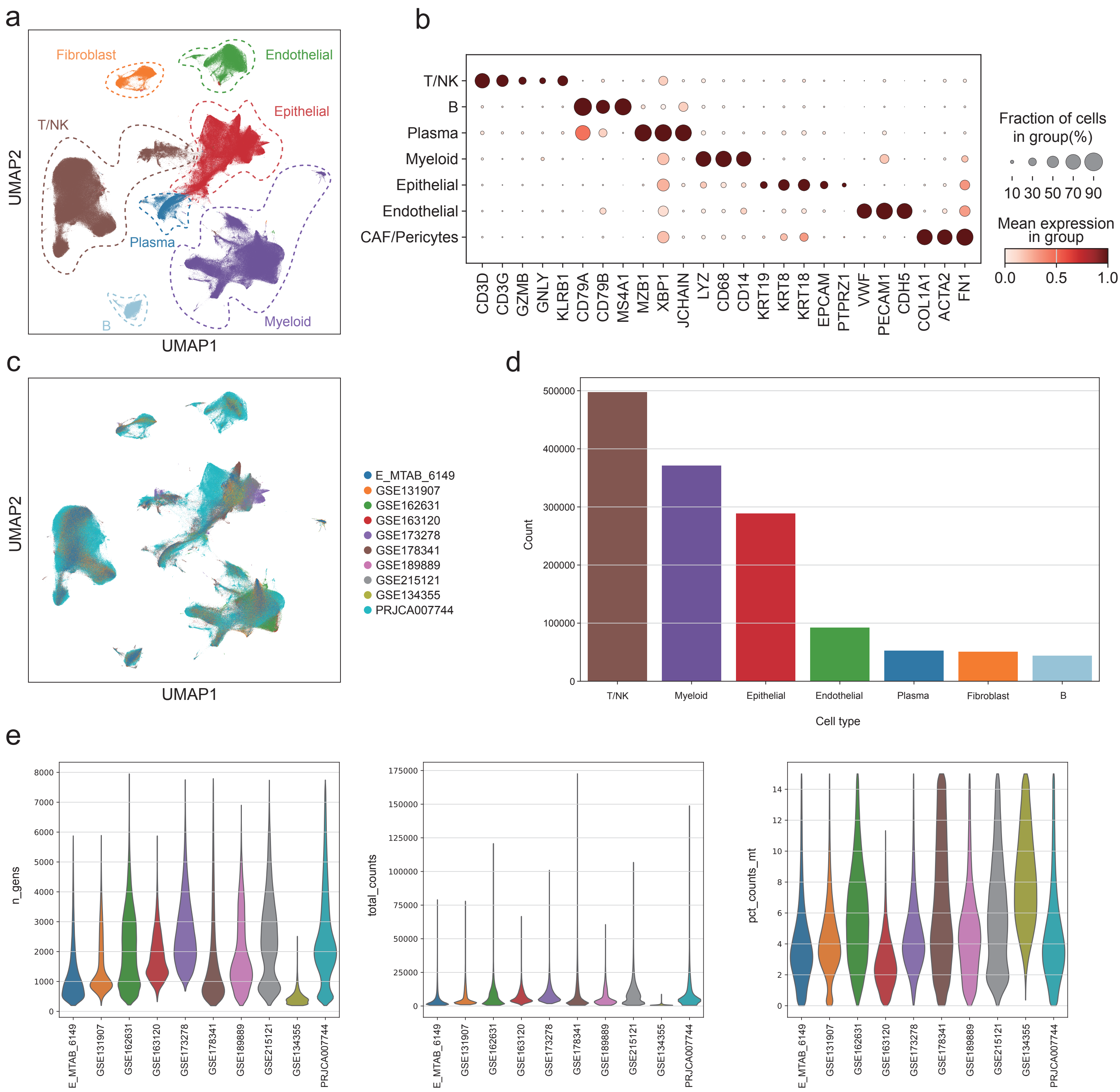

Fig. S1 | scRNA-seq profiling of 10 data sets across five human cancer types.

a. Uniform Manifold Approximation and Projection (UMAP) colour-coded by the seven broad cell types.

b. Bubble plot showing highly expressed marker genes specific to each major cell type.

c. UMAP representing all cell populations and their distribution across the studies.

d. Bar plot showing cell count distribution per cell type.

e. Violin plot showing quality control metrics per study, including: genes per cell (n_genes), total transcripts per cell (total_counts), mitochondrial content percentage (pct_counts_mt).

Fig.S2

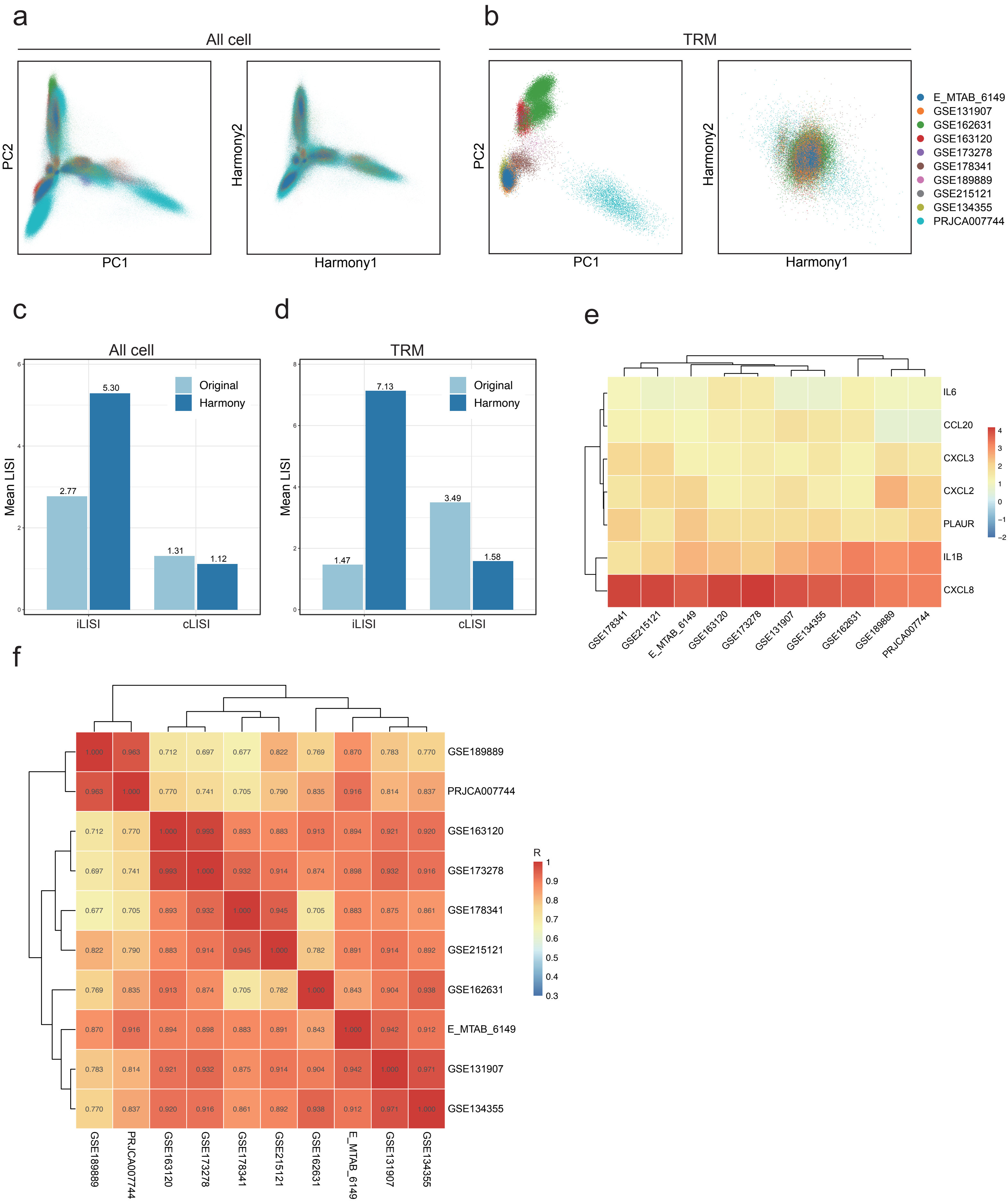
a
All cell
PC2
PC1
Harmony2
Harmony1
b
TRM
PC2
PC1
Harmony2
Harmony1
E_MTAB_6149
GSE131907
GSE162631
GSE163120
GSE173278
GSE178341
GSE189889
GSE215121
GSE134355
PRJCA007744
c
All cell
Mean LISI
Original
Harmony
2.77
5.30
1.31
1.12
iLISI
cLISI
d
TRM
Mean LISI
Original
Harmony
1.47
7.13
3.49
1.58
iLISI
cLISI
e
IL6
CCL20
CXCL3
CXCL2
PLAUR
IL1B
CXCL8
GSE178341
GSE215121
E_MTAB_6149
GSE163120
GSE173278
GSE131907
GSE134355
GSE162631
GSE189889
PRJCA007744
f
GSE189889 1.000 0.963 0.712 0.697 0.677 0.822 0.769 0.870 0.783 0.770
PRJCA007744 0.963 1.000 0.770 0.741 0.705 0.790 0.835 0.916 0.814 0.837
GSE163120 0.712 0.770 1.000 0.993 0.893 0.883 0.913 0.894 0.921 0.920
GSE173278 0.697 0.741 0.993 1.000 0.932 0.914 0.874 0.898 0.932 0.916
GSE178341 0.677 0.705 0.893 0.932 1.000 0.945 0.705 0.883 0.875 0.861
GSE215121 0.822 0.790 0.883 0.914 0.945 1.000 0.782 0.891 0.914 0.892
GSE162631 0.769 0.835 0.913 0.874 0.705 0.782 1.000 0.843 0.904 0.938
E_MTAB_6149 0.870 0.916 0.894 0.898 0.883 0.891 0.843 1.000 0.942 0.912
GSE131907 0.783 0.814 0.921 0.932 0.875 0.914 0.904 0.942 1.000 0.971
GSE134355 0.770 0.837 0.920 0.916 0.861 0.892 0.938 0.912 0.971 1.000
R
GSE189889
PRJCA007744
GSE163120
GSE173278
GSE178341
GSE215121
GSE162631
E_MTAB_6149
GSE131907
GSE134355

Fig. S2 | Batch effect control between different datasets.

a-b. Principal Component Analysis (PCA) visualization illustrating the batch effect distribution across samples before and after Harmony integration.

c-d. Bar plot illustrating the change in Local Inverse Simpson's Index (LISI) before and after Harmony integration. iLISL: integration LISI; cLISI: correction LISI.

e. Heatmap demonstrating the expression consistency of key genes across TRM subsets between batches.

f. Heatmap depicting Pearson correlation coefficients between batches, calculated based on key gene expression within TRM subsets.

Fig.S3

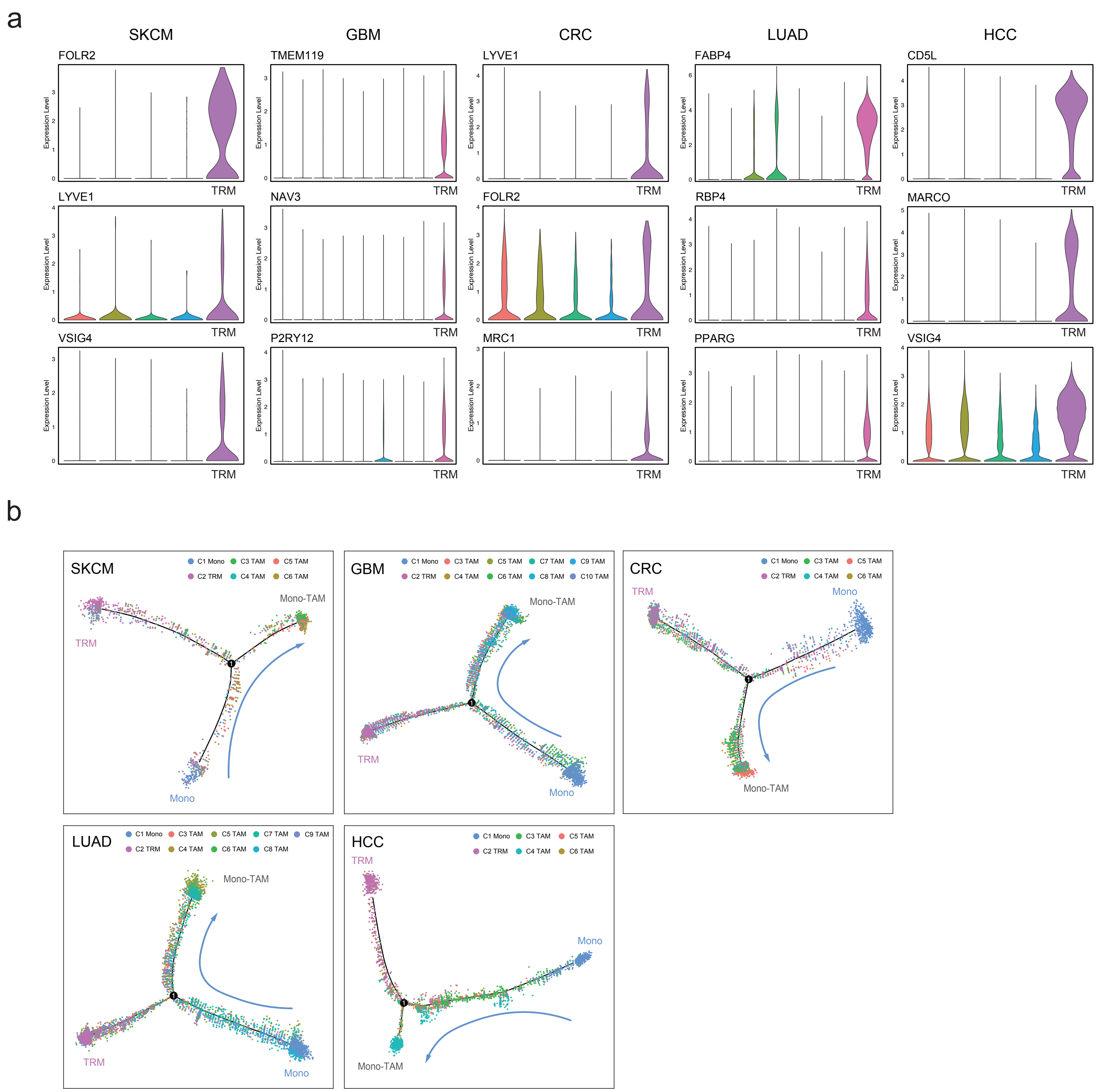

Fig. S3 | Identification of tissue-resident macrophages (TRM) across various tissues.

a. Violin plots showing uniquely enriched marker genes of TRM across tissues.

b. Pseudotime trajectory plot showing divergent developmental paths of TRM versus monocyte-derived tumor-associated macrophages (TAM).

Fig.S4

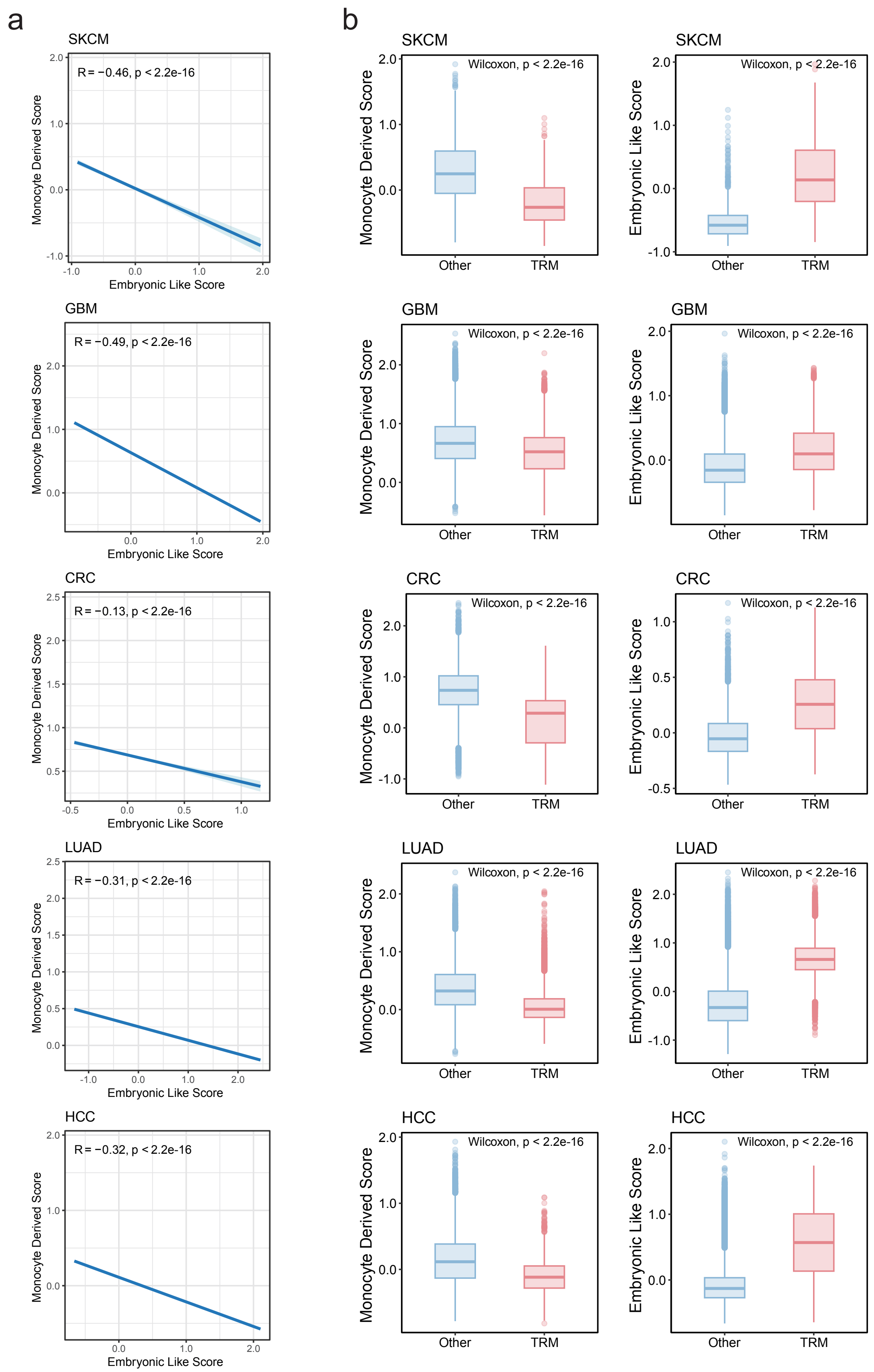

Fig. S4 | Embryo-related characteristics of tissue-resident macrophages (TRM).

a. Correlation between embryonically-derived and monocyte-derived signatures in tumor-associated macrophages (TAM, including TRM-TAM and Mono-TAM) (Pearson's r).

b. Boxplot comparing embryonic-derived and monocyte-derived signature scores between TRM and other TAM. Statistical significance was assessed using the Wilcoxon rank-sum test.

Fig.S5

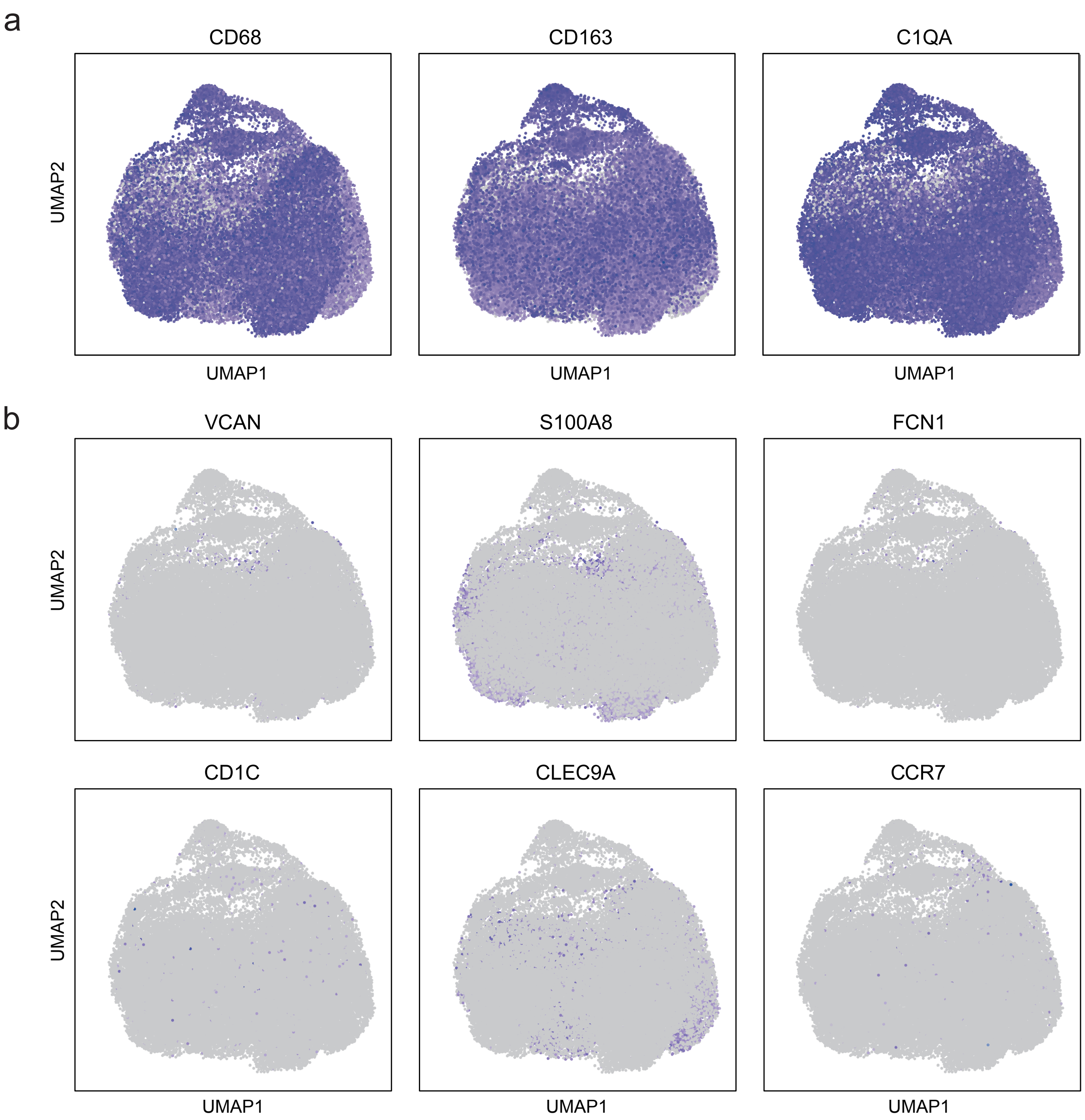

Fig. S5 | Expression patterns of canonical myeloid markers in TRM subpopulations.

a. Expression of macrophage markers across TRM subpopulations.

b. Expression of monocyte and dendritic markers across TRM subpopulations.

Fig.S6

a

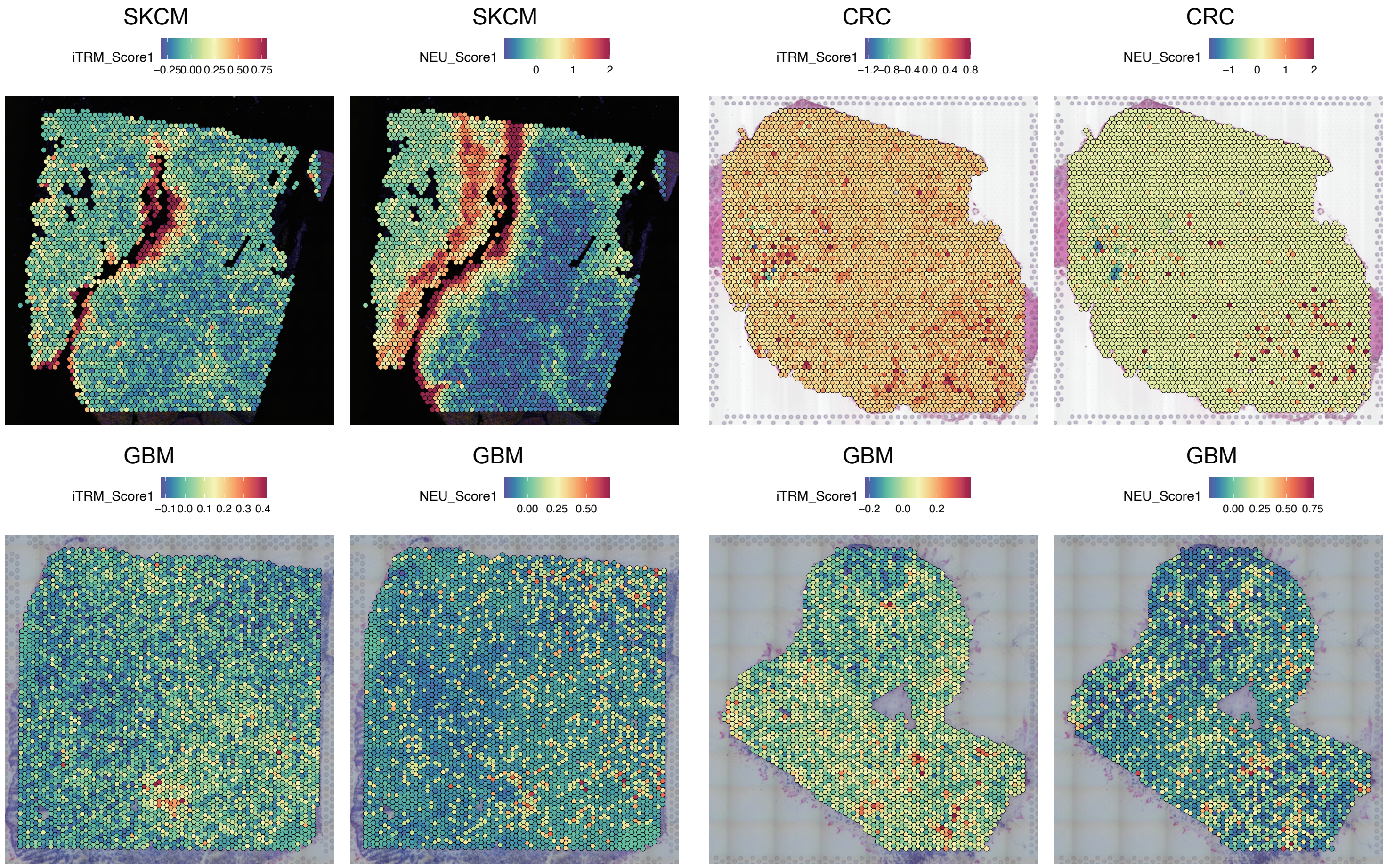

Fig. S6 | Spatial proximity of inflammatory tissue-resident macrophages (iTRM) and neutrophils across tissue niches.

a. Spatial co-expression landscapes of iTRM and neutrophil signatures across malignancies.

Fig.S7

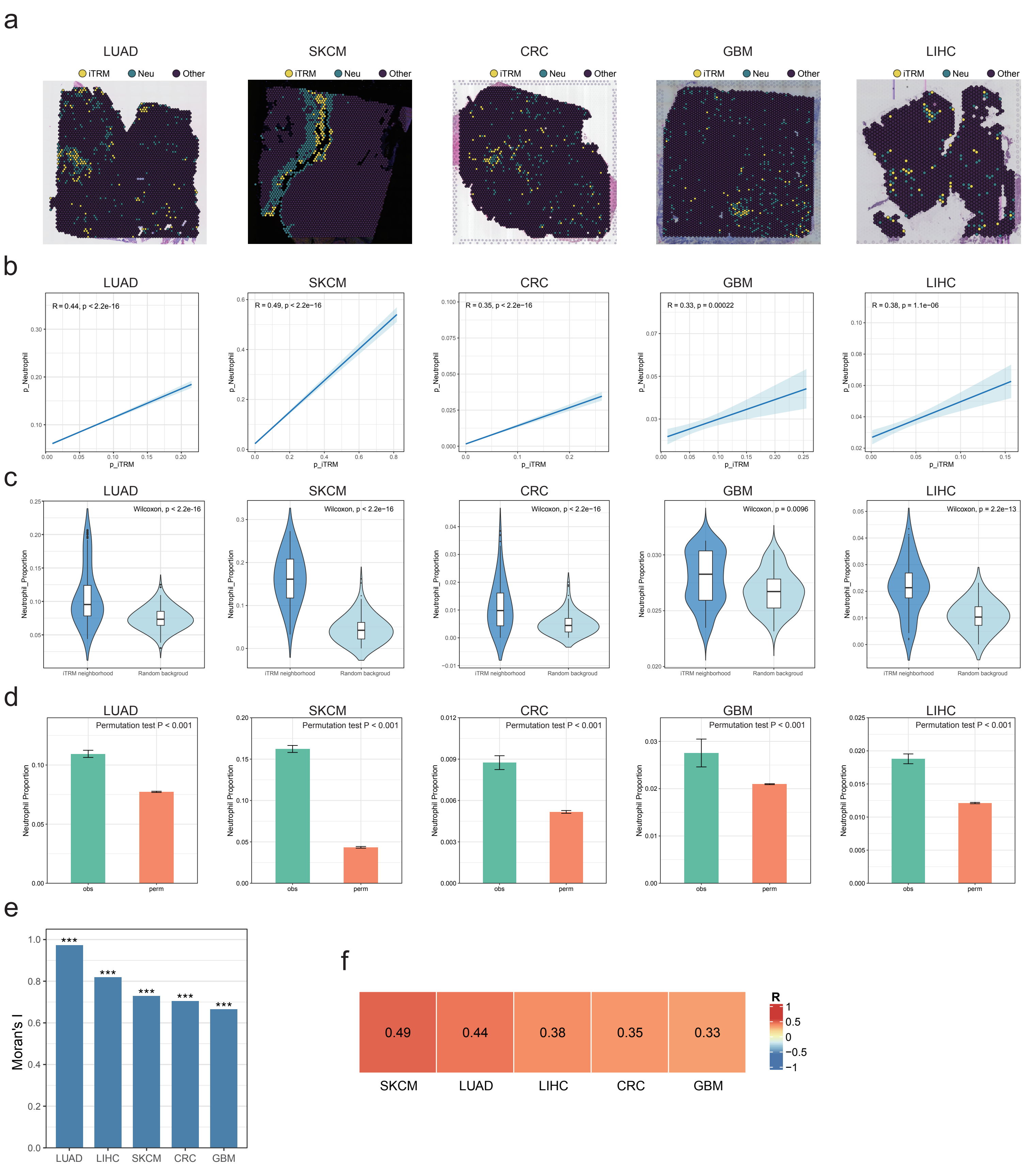
a
LUAD
SKCM
CRC
GBM
LIHC
iTRM
Neu
Other
b
R = 0.44, p < 2.2e-16
R = 0.49, p < 2.2e-16
R = 0.35, p < 2.2e-16
R = 0.33, p = 0.00022
R = 0.38, p = 1.1e-06
p_Neutrophil
p_iTRM
c
Wilcoxon, p < 2.2e-16
Wilcoxon, p < 2.2e-16
Wilcoxon, p < 2.2e-16
Wilcoxon, p = 0.0096
Wilcoxon, p = 2.2e-13
Neutrophil_Proportion
iTRM neighborhood
Random backgroud
d
Permutation test P < 0.001
Neutrophil Proportion
obs
perm
e
Moran's I
***
LUAD
LIHC
SKCM
CRC
GBM
f
R
0.49
0.44
0.38
0.35
0.33
SKCM
LUAD
LIHC
CRC
GBM

Fig. S7 | Spatial transcriptomic features of iTRM.

a. Spatial co-localization of C3_iTRM_IL1B and neutrophils across various cancer types revealed by spot deconvolution.

b. Spatial correlation between C3_iTRM_IL1B and neutrophil proportions based on spot deconvolution. Pearson's method was used to calculate correlation coefficients. p_iTRM: proportion of C3_iTRM_IL1B per spot; p_Neutrophil: proportion of neutrophils per spot.

c. Spatial neighborhood analysis revealed significant enrichment of neutrophils in the vicinity of iTRM compared to random background.

d. Bar chart showing the true mean proportion of neutrophils within iTRM spot neighborhoods (obs) and the mean proportion after random permutation (perm).

e. Bar chart showing Moran's I calculation results across cancer types.

f. The heatmap showing differential spatial correlations between iTRM and neutrophils across diverse cancer types.

Fig.S8

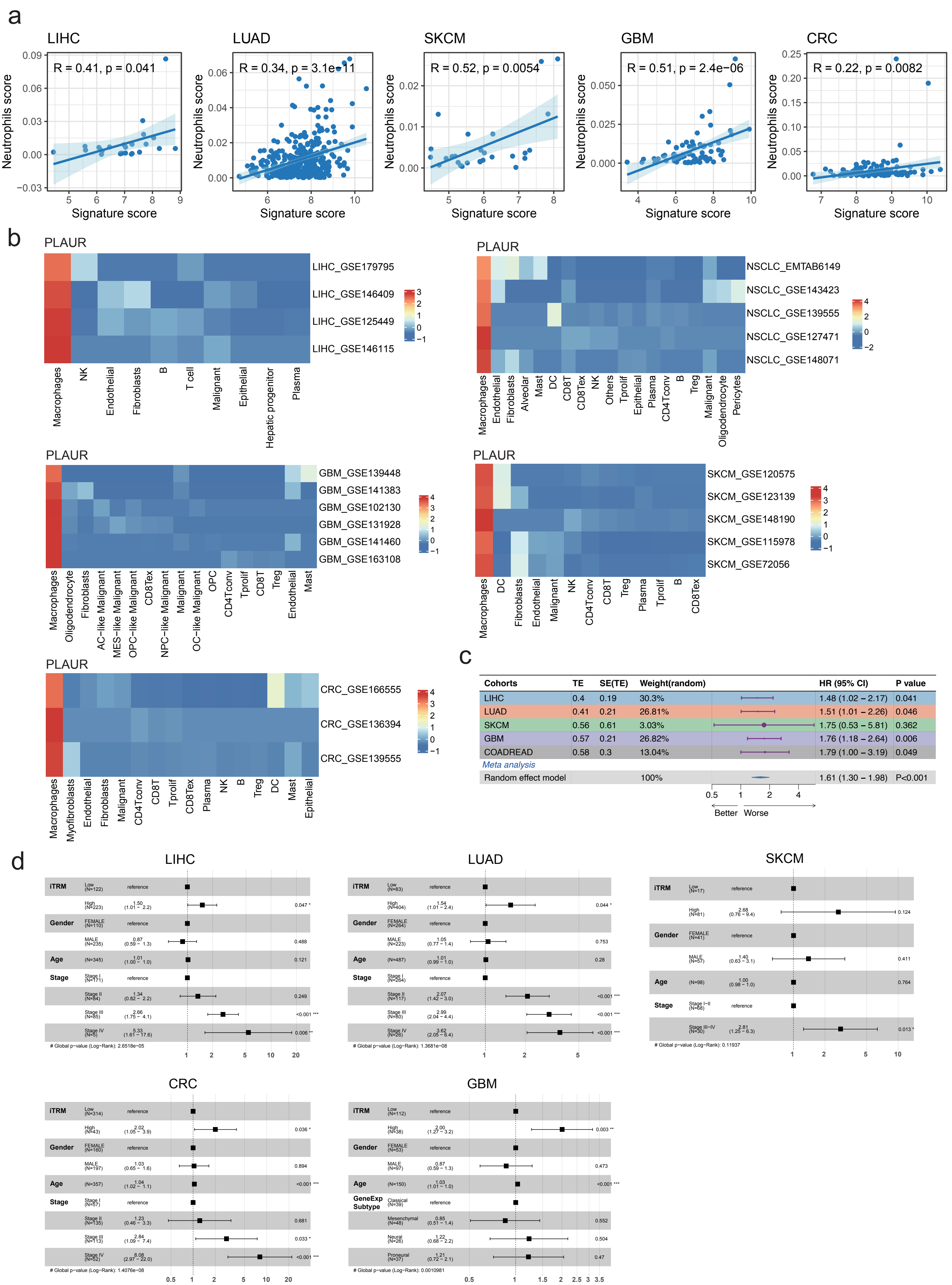
a
LIHC
R = 0.41, p = 0.041
LUAD
R = 0.34, p = 3.1e−11
SKCM
R = 0.52, p = 0.0054
GBM
R = 0.51, p = 2.4e−06
CRC
R = 0.22, p = 0.0082
Neutrophils score
Signature score
b
PLAUR
LIHC_GSE179795
LIHC_GSE146409
LIHC_GSE125449
LIHC_GSE146115
Macrophages
NK
Endothelial
Fibroblasts
B
T cell
Malignant
Epithelial
Hepatic progenitor
Plasma
NSCLC_EMTAB6149
NSCLC_GSE143423
NSCLC_GSE139555
NSCLC_GSE127471
NSCLC_GSE148071
Alveolar
Mast
DC
CD8T
CD8Tex
Others
Tprolif
CD4Tconv
Treg
Oligodendrocyte
Pericytes
GBM_GSE139448
GBM_GSE141383
GBM_GSE102130
GBM_GSE131928
GBM_GSE141460
GBM_GSE163108
AC−like Malignant
MES−like Malignant
OPC−like Malignant
NPC−like Malignant
OC−like Malignant
OPC
SKCM_GSE120575
SKCM_GSE123139
SKCM_GSE148190
SKCM_GSE115978
SKCM_GSE72056
CRC_GSE166555
CRC_GSE136394
CRC_GSE139555
Myofibroblasts
c
Cohorts | TE | SE(TE) | Weight(random) | HR (95% CI) | P value
LIHC | 0.4 | 0.19 | 30.3% | 1.48 (1.02 – 2.17) | 0.041
LUAD | 0.41 | 0.21 | 26.81% | 1.51 (1.01 – 2.26) | 0.046
SKCM | 0.56 | 0.61 | 3.03% | 1.75 (0.53 – 5.81) | 0.362
GBM | 0.57 | 0.21 | 26.82% | 1.76 (1.18 – 2.64) | 0.006
COADREAD | 0.58 | 0.3 | 13.04% | 1.79 (1.00 – 3.19) | 0.049
Meta analysis
Random effect model | 100% | 1.61 (1.30 – 1.98) | P<0.001
Better
Worse
d
LIHC
LUAD
SKCM
CRC
GBM
iTRM
Gender
Age
Stage
GeneExp Subtype
reference
Global p-value (Log-Rank): 2.6518e−05
Global p-value (Log-Rank): 1.3681e−08
Global p-value (Log-Rank): 0.11937
Global p-value (Log-Rank): 1.4076e−08
Global p-value (Log-Rank): 0.0010981

Fig. S8 | The functional role of iTRM and its association with prognosis.

a. Scatter plots showing the correlation between the C3_iTRM_IL1B signature and neutrophil infiltration across cancer types (correlation coefficient $R > 0.3$, $p < 0.05$ indicates significant positive association).

b. Heatmap showing the specific expression of CD87 (PLAUR) in macrophages across diverse cancer types.

c. Forest plot showing effects of iTRM signatures across various cancer types calculated using random-effects model.

d. Forest plot presents the hazard ratios (HR) of various factors derived from multivariate COX regression analyses across different cancer types.

Fig.S9

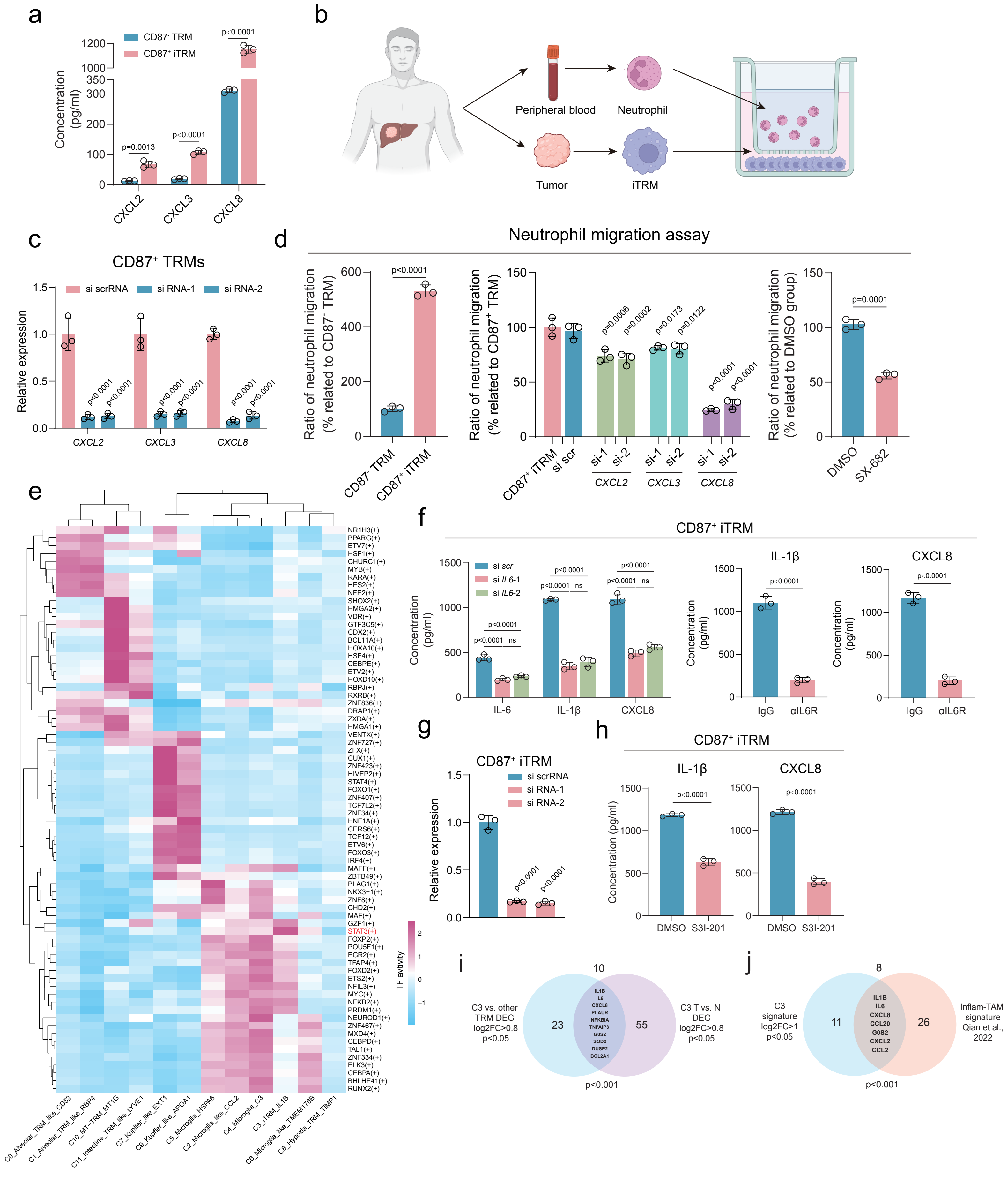
a
CD87⁻ TRM
CD87⁺ iTRM
Concentration (pg/ml)
CXCL2
CXCL3
CXCL8
b
Peripheral blood
Neutrophil
Tumor
iTRM
c
CD87⁺ TRMs
si scrRNA
si RNA-1
si RNA-2
Relative expression
d
Neutrophil migration assay
Ratio of neutrophil migration (% related to CD87⁻ TRM)
Ratio of neutrophil migration (% related to CD87⁺ TRM)
Ratio of neutrophil migration (% related to DMSO group)
CD87⁻ TRM
CD87⁺ iTRM
si scr
DMSO
SX-682
e
TF avtivity
STAT3(+)
f
CD87⁺ iTRM
si scr
si IL6-1
si IL6-2
IL-6
IL-1β
CXCL8
IgG
αIL6R
g
CD87⁺ iTRM
h
CD87⁺ iTRM
DMSO
S3I-201
i
C3 vs. other TRM DEG log2FC>0.8 p<0.05
C3 T vs. N DEG log2FC>0.8 p<0.05
p<0.001
j
C3 signature log2FC>1 p<0.05
Inflam-TAM signature Qian et al., 2022
p<0.001

Fig. S9 | Functional Validation of iTRM Phenotypic Regulation and Neutrophil Crosstalk in Inflammatory Responses.

a. ELISA quantification of CXCL2, CXCL3, and CXCL8 concentrations in culture supernatants from CD87- TRMs and CD87+ iTRMs. p value by Student's t test; Experiment was repeated three times. A p value of less than 0.05 indicates a statistical difference. Error bar represent mean ± SEM.

b. The experimental design of the transwell neutrophil migration assay.

c. CXCL2, CXCL3 and CXCL8 knocked-down efficiency verification of CD87+TRMs by RT-qPCR. p values are calculated relative to si scrRNA

d. Quantification of neutrophil migration ratio in CD87+/- TRMs (left), or after indicated genes knockdown in CD87+ TRM (middle) , or after SX-682 treatment of neutrophils (right). p values in the middle panel is calculated relative to si scrRNA.

(d, left and right) p value by Student's t test; (c, d, middle) p value by one-way ANOVA; (c-d) Experiment was repeated three times. A p value of less than 0.05 indicates a statistical difference. Error bar represent mean ± SEM unless otherwise indicated.

e. Heatmap displaying transcription factor (TF) activities inferred by pySCENIC for each TRM subset.

f. ELISA quantification of IL-6, IL-1β and CXCL8 in supernatants from CD87+ iTRMs after IL6 knockdown (si IL6-1/si IL6-2) or IL6R blockade (αIL6R) as indicated.

g. IL6 knockdown efficiency verification of CD87+ TRMs by RT-qPCR. p values are calculated relative to si scrRNA.

h. ELISA quantification of IL-1β and CXCL8 in supernatants from CD87+ iTRMs treated with the STAT3 inhibitor S3I-201.

i. Venn diagram showing overlap between C3_iTRM_IL1B subpopulation-enriched genes and tumor-specific highly expressed genes within C3_iTRM_IL1B cells (p-value calculated via hypergeometric test).

j. Venn diagram showing a significant high correlation between C3_iTRM_IL1B and Inflam-TAM phenotype.

(f, middle and right, h) p value by Student's t test; (f, left, g) p value by one-way ANOVA. (f-h) Experiment was repeated three times. A p value of less than 0.05 indicates a statistical difference. Error bar represent mean ± SEM.

Fig.S10

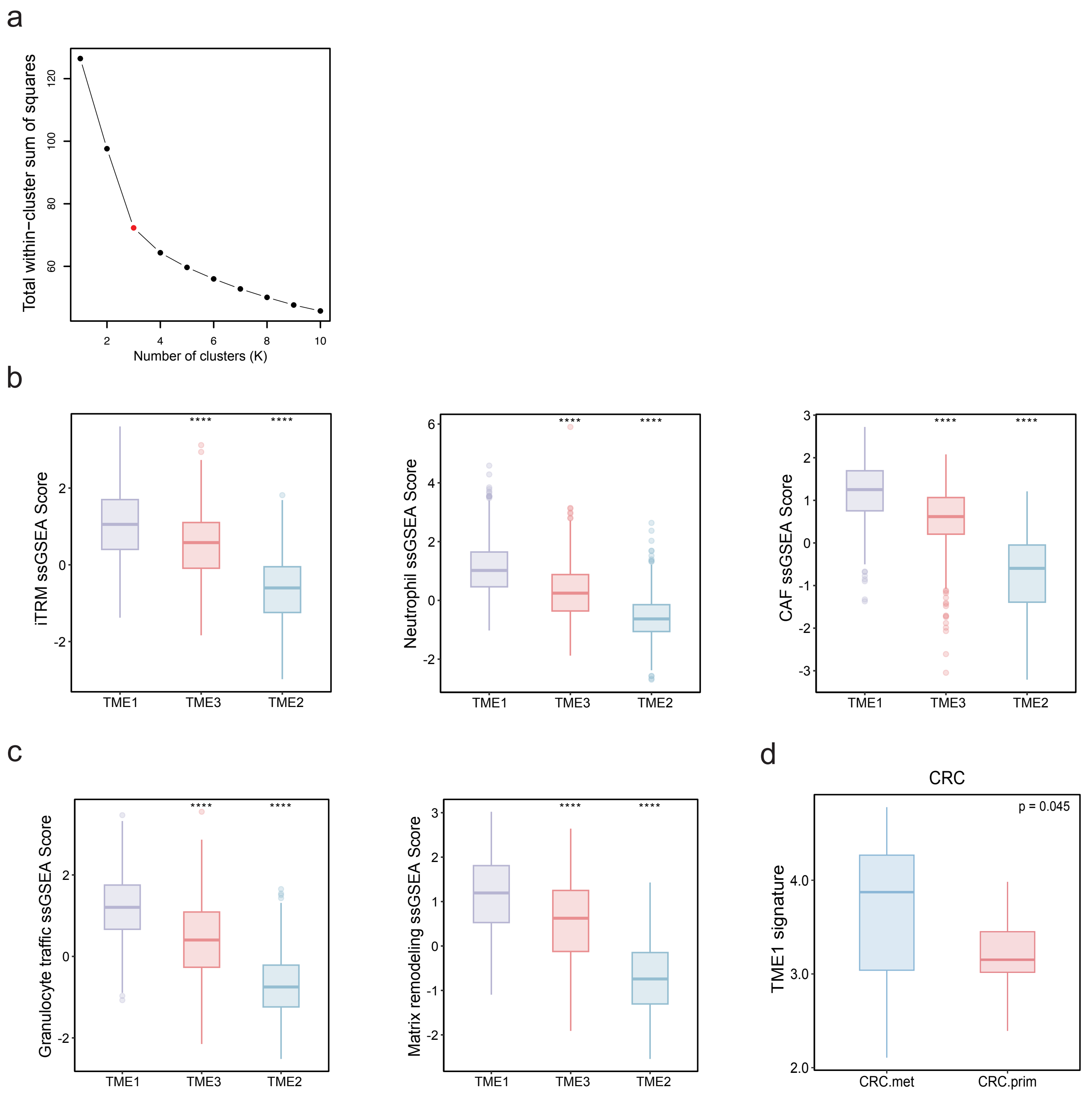

Fig. S10 | Identification of iTRM-specific tumor microenvironment (iTRM-TME).

a. Determination of optimal cluster number (K) using the elbow method. The plot shows the total within-cluster sum of squares (WSS) for K-means clustering across candidate K values (K=1 to 10). The "elbow point" (K=3) suggests the optimal balance between model complexity and explanatory power.

b. Box plots showing the enrichment of iTRMs, neutrophils, and tumor-associated fibroblasts (CAF) in different TME subtypes.

c. Box plots showing functional enrichment of granulocyte traffic and matrix remodeling in different TME subtypes.

d. Box plot showing that TME1 (iTRM-TME) is significantly associated with tumor metastasis in colorectal cancer (CRC).

Fig.S11

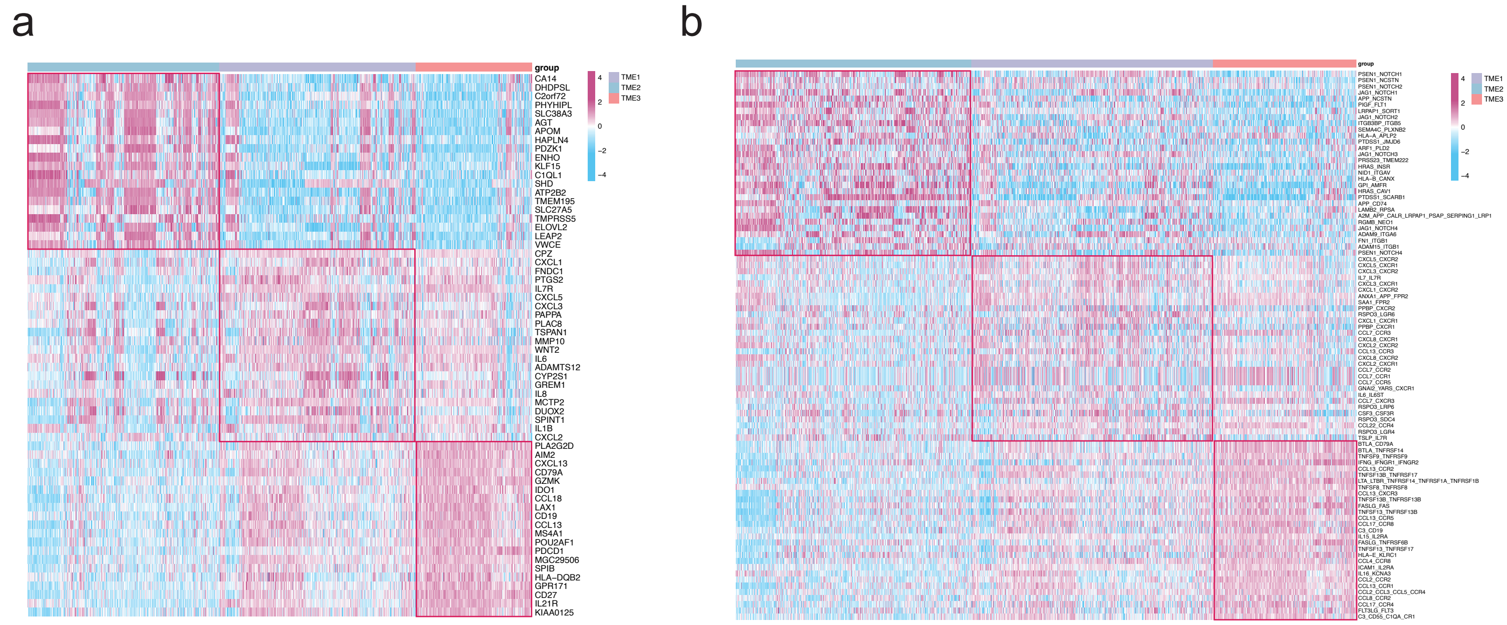

Fig. S11 | Characteristics of iTRM-enriched tumor immune microenvironment.

a. Heatmap displaying differentially expressed genes across TME subtypes.

b. Heatmap showing differential receptor-ligand pair expression patterns among TME subtypes.

Fig.S12

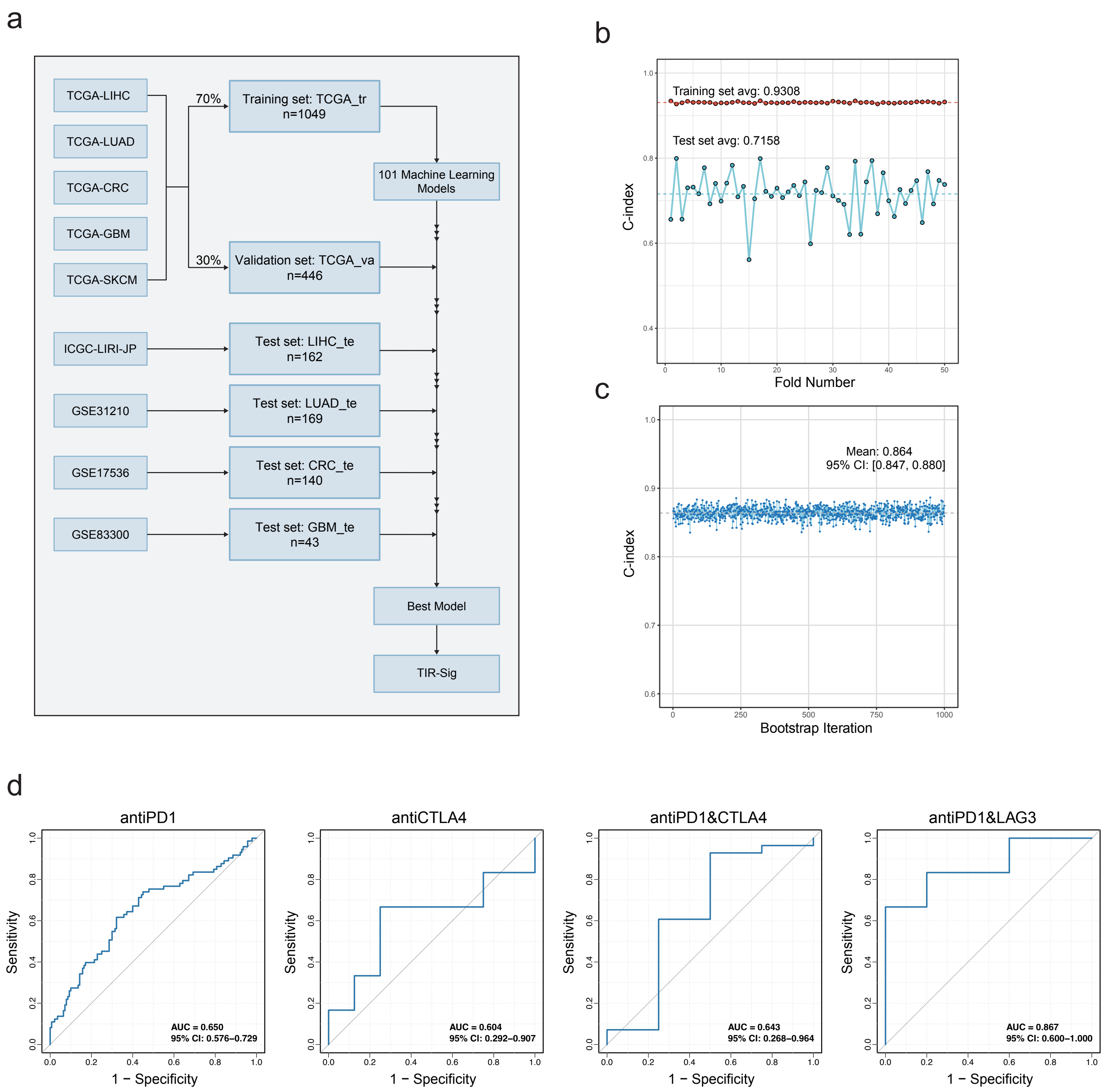

Fig. S12 | Construction of TRM inflammatory remodeling signature (TIR-Sig).

a. Pipeline for constructing IRT-Sig via ensemble learning with 101 ML models.

b. The line chart showing the changes in the C-index for the training set and test set during the 10-fold 5-repeat cross-validation process.

c. The line chart showing the changes in the C-index for the training set during the 1000-iteration Bootstrap validation process.

d. ROC curve of the TRM inflammatory remodeling signature (TIR-Sig) for predicting differential responses to immune checkpoint inhibitor (ICI) therapy across different immunotherapy in ICI-treated cohorts.

Fig.S13

a

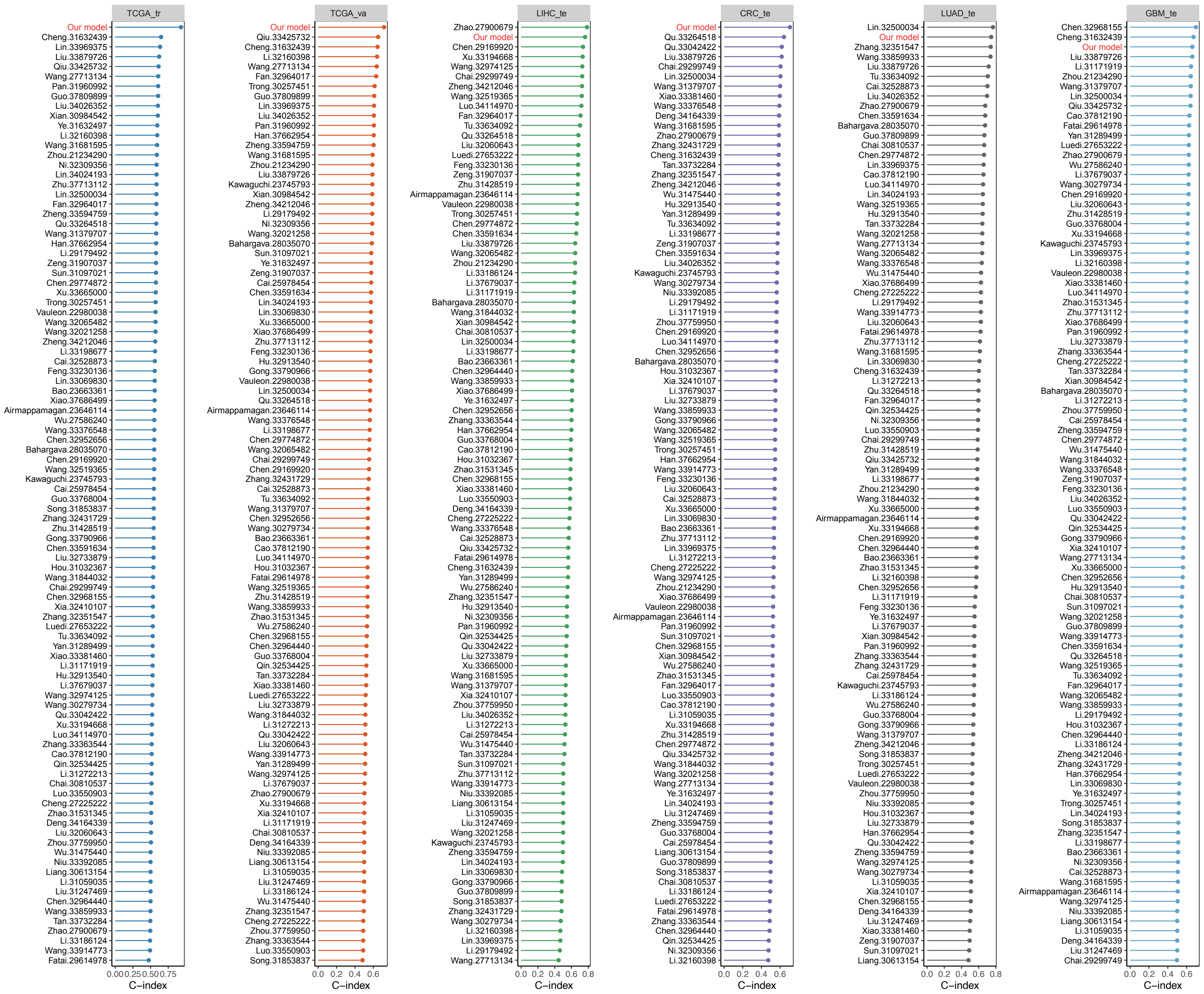

b

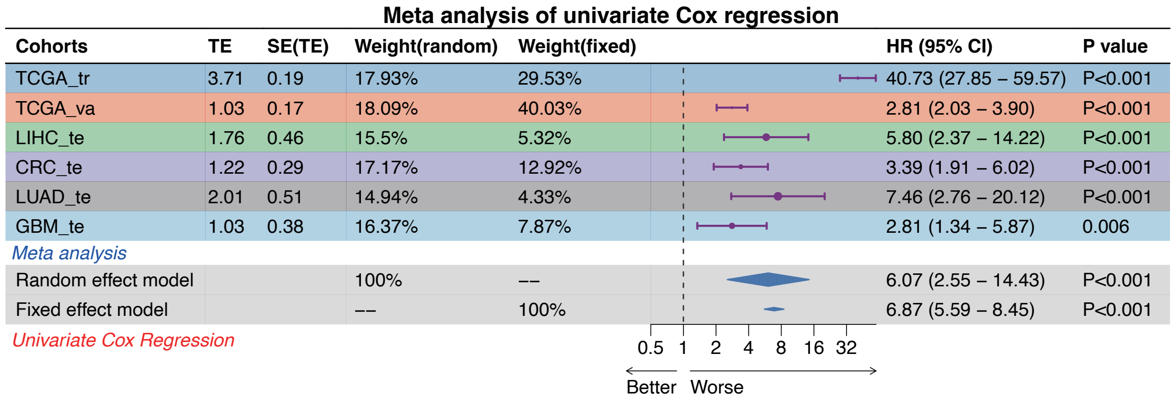

Fig. S13 | Performance Evaluation of TRM inflammatory remodeling signature (TIR-Sig).

a. Benchmark comparison of TIR-Sig against 95 published signatures in training, validation, and test cohorts.

b. Prognostic hazard ratios (HR) of TIR-Sig across training, validation, and test cohorts.
